# Electron Transfer‐Tailored D‐Band Center to Boost Nanozyme Catalysis for Interpretable Machine Learning‐Empowered Intelligent Biosensing

**DOI:** 10.1002/advs.202505712

**Published:** 2025-08-23

**Authors:** Yuechun Li, Chenxin Ji, Zhaowen Cui, Jianxing Feng, Liang Zhang, Sha Liu, Wentao Zhang, Yanwei Ji, Yizhong Shen, Jianlong Wang

**Affiliations:** ^1^ College of Food Science and Engineering Northwest A&F University Yangling Shaanxi 712100 China; ^2^ School of Food & Biological Engineering Hefei University of Technology Hefei 230009 China

**Keywords:** AIEgens, D‐band center, electron transfer, immunoassay, machine learning, nanozyme, pathogens, SHAP

## Abstract

The escalating global burden of infectious diseases demands biosensing technologies that transcend the complexity‐sensitivity‐accuracy trade‐off in real‐world applications. Herein, an interpretable machine learning‐empowered multimodal biosensor synergizing electron transfer‐enhanced nanozymes and aggregation‐induced emission luminogens (AIEgens) for ultrasensitive pathogen detection is presented. By engineering aminophenol formaldehyde resin nanobowls anchored with monodisperse Pt nanoparticles, interfacial electron transfer (N→Pt→O) induces an upshift of Pt d‐band center relative to the Fermi level, as validated by density functional theory. This electronic modulation optimizes H_2_O_2_ adsorption energy, lowers the energy barrier of the rate‐determining step, and reduces activation energy, resulting in a 3.4‐fold enhancement in peroxidase‐like activity over conventional Pt nanozymes. Then, AIEgens are strategically integrated to generate cross‐validated anti‐interference signals, achieving a record‐low detection limit for *Salmonella typhimurium*, surpassing classical immunoassays in sensitivity and accuracy. A SHapley Additive exPlanations (SHAP)‐guided eXtreme Gradient Boosting (XGBoost) algorithm dynamically fuses multimodal signals, enhancing sensitivity by five fold over single‐mode detection and delivering 100% diagnostic accuracy for positive samples. SHAP further deciphers the synergetic mechanism, revealing concentration‐dependent signal contributions and validating decision logic. This work pioneers a nanozyme‐AI co‐design framework, bridging d‐band‐driven catalytic precision and machine learning‐powered signal intelligence to redefine biosensing paradigms for combating public health emergencies.

## Graphical abstract

This study pioneers a rational nanozyme design strategy guided by electron transfer mechanisms while advancing biosensing paradigms through interpretable machine learning‐enhanced signal intelligence. By transforming biosensors from passive transducers to active diagnostic systems, this work establishes a versatile framework for addressing global health crises through synergistic nanomaterial engineering and artificial intelligence.

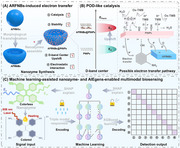

## 1 Introduction

Pathogenic threats, exemplified by *Salmonella typhimurium* (*S. typhimurium*), contribute to over 600 million global infections with 420 000 annual fatalities, imposing catastrophic burdens on public health systems (WHO, 2023).^[^ ^1^ ^]^ Current biosensors, however, remain trapped in a “complexity‐sensitivity‐accuracy” trilemma. Nanozymes, as artificial enzyme mimics, hold immense potential for biosensing of pathogens due to their tunable catalytic activity and robustness in harsh environments.^[^ ^2^ ^]^ However, the existing research ignored two core bottlenecks: nanozyme activity and multisignal crosstalk. The insufficient catalytic activity of nanozymes has strictly restrained the development of nanozyme‐based biosensors.^[^ ^3^ ^]^ For example, the catalytic inefficiency of noble metal nanozymes, such as Pt nanoparticles (PtNPs), stems from their unoptimized d‐band center (E_d_) relative to the Fermi level (E_F_), which governs substrate adsorption and intermediate stabilization.^[^ ^4^ ^]^ A large number of researches demonstrated electron transfer could be occurred between nanozymes and supports to tailor the electronic structure of nanozymes, indicating that the optimization of the d‐band center of nanozymes by interfacial electron transfer between nanozymes and supports is a feasible method.^[^ ^5^ ^]^ The cross‐modal signal crosstalk can cause cross‐interference and is difficult to achieve cross‐validation of multiple signals, which seriously reduces the detection reliability. Concurrently, machine learning promises to fuse multimodal signals, but “black‐box” models obscure decision logic, hindering trust in clinical settings and ignoring the dynamic interaction effect of multisignal.

**Scheme 1:**
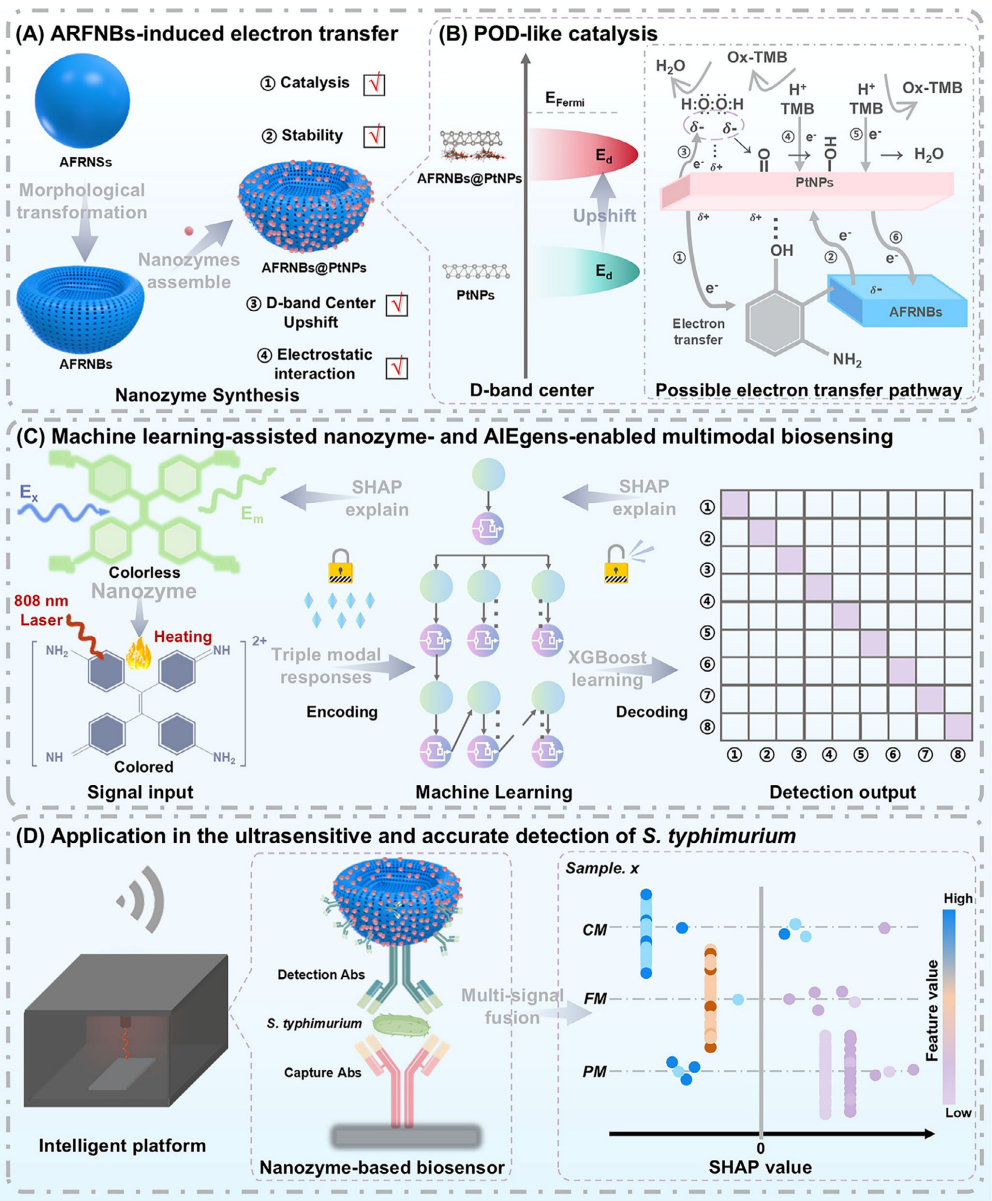
A,B) The development of AFRNBs@PtNPs nanozymes with the favored electron transfer pathway, C) XGBoost learning algorithm‐assisted nanozyme‐based multisignal biosensors, and D) the SHAP interpretable frame analysis‐driven feature optimization in biosensing.

Herein, we proposed a collaborative innovation strategy of “electron engineering‐signal cross‐validated‐intelligent decoding” and constructed a new generation of intelligent biosensing platform for the detection of pathogens (**Scheme** **1**). First, monodisperse PtNPs were accurately anchored by an aminophenol‐formaldehyde resin nanobowl (AFRNBs) and exhibited the interfacial electron transfer effect between nanozyme and support (N→Pt→O). Density functional theory (DFT) calculations revealed that AFRNBs could upshift the position of E_d_ of PtNPs relative to E_F_. This electronic modulation driven by N to Pt electron donation and Pt to O back‐donation enhances the adsorption energy of H_2_O_2_ and oxygen intermediate, lowers the energy barrier of the rate‐determining step (RDS), and reduces reaction activation energy (*E_a_*), thus greatly boosting the peroxidase (POD)‐like catalysis. Second, tetrakis(4‐aminophenyl)ethene (TPEN) aggregation‐induced emission luminogens (AIEgens) were innovatively introduced to enable anti‐interference cross‐validated signals (colorimetric λ_A_ = 600 nm, fluorescent e_m_ = 525 nm, and photothermal η = 39.81%), overcoming the low sensitivity and accuracy of classical immunoassay. Furthermore, eXtreme Gradient Boosting (XGBoost)‐SHapley Additive exPlanations (SHAP) interpretable frame learning algorithm was used to dynamically optimize and fuse the multisignal, which could not only help the improvement of the detection sensitivity and accuracy, but also decode the weight of concentration‐dependent signals and provide a data‐driven basis for feature optimization in biosensing. This study not only established a new paradigm for the rational design of nanozyme, but also created a new system of “material‐signal‐algorithm” full chain interpretable biosensor, marking a new era of biosensor technology from “passive response” to “active decision‐making” and providing a key technical reserve for global public health security.

## 2 Results and Discussion

### 2.1 Synthesis and Characterization of AFRNBs@PtNPs

**Figure 1:**
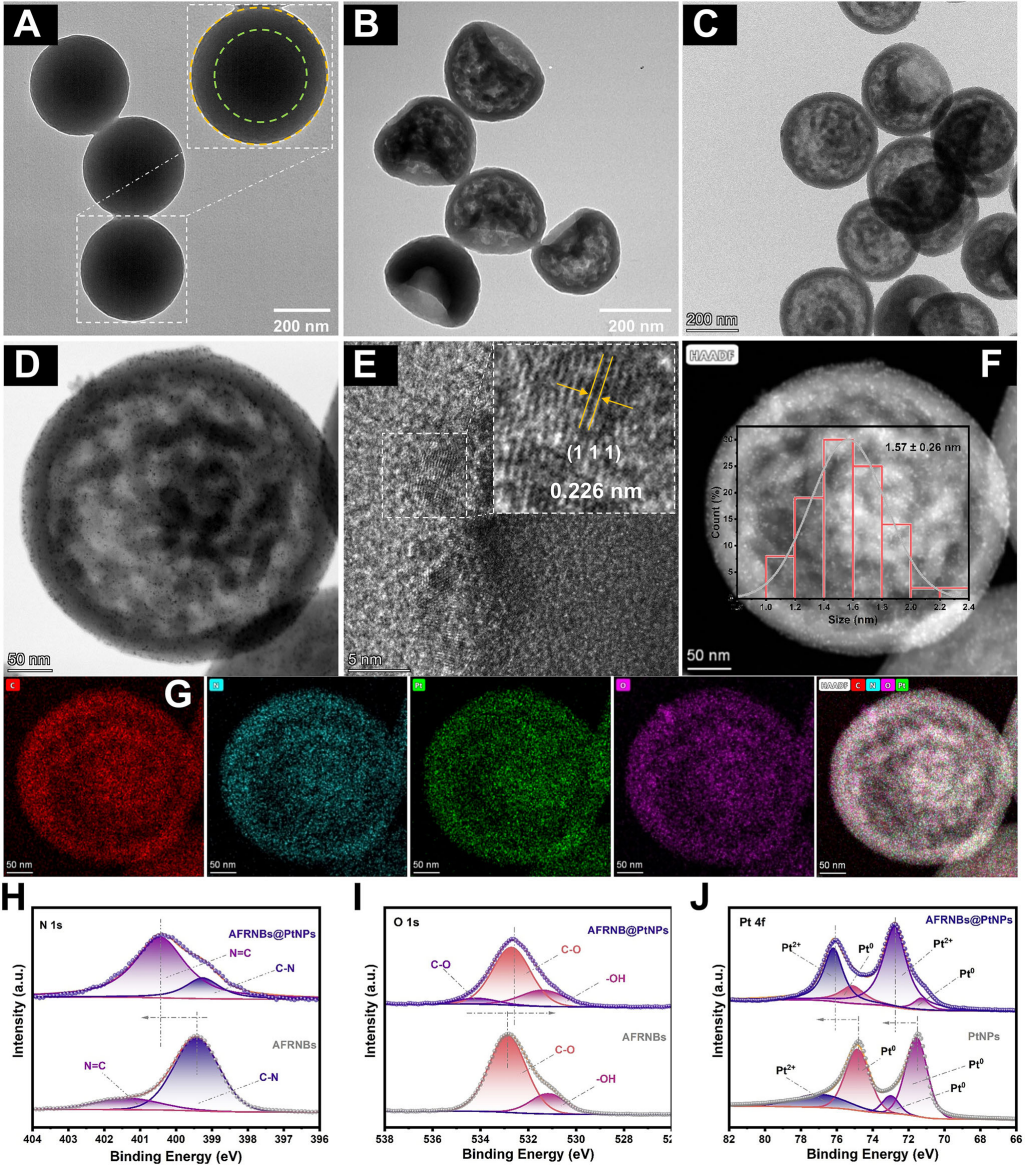
A) TEM image of AFR, B) AFRNBs, C) AFRNBs@PtNPs at wide visual field, D) AFRNBs@PtNPs at narrow visual field, E) HRTEM of AFRNBs@PtNPs, F) AFRNBs@PtNPs under HADDF mode, G) EDS mapping of AFRNBs@PtNPs, H) N 1s spectra, I) O 1s spectra, J) and Pt 4f spectra.

AFRNBs@PtNPs were synthesized by an in situ reduction strategy of monodisperse PtNPs on the well‐dispersed AFRNBs. First, the sphere‐like aminophenol‐formaldehyde resin (AFR) was synthesized, and its morphology was observed by transmission electron microscope (TEM), which showed uniform and monodisperse sphere‐like nanoparticles, whose monodispersity was favorable for the morphological post‐modification. As exhibited in **Figure** **1**A, the polymerization degree of the prepared AFR was inhomogeneous between the shell and core because of steric hindrance during the formation of the AFR precursor, which paved the way for etching the morphology of AFR into hollow nanostructure through dissolution‐repolymerization tailoring strategy.^[^ ^6^ ^]^ Subsequently, anhydrous ethanol was used to transport the morphology from sphere‐like AFR to AFRNBs via the inhomogeneous dissolution of AFR in ethanol, which tended to provide more anchoring sites for PtNPs, and the hollow and bowl‐like nanostructure of AFRNBs could be clearly observed in Figure 1B. Afterward, PtNPs were precisely anchored on the AFRNBs to form AFRNBs@PtNPs with the help of ascorbic acid under 80 °C water bath. By virtue of the reducibility‐powered the Pt^4+^ to Pt^0^, PtNPs were uniformly synthesized on each AFRNB. As displayed in Figure 1C,D by different magnifications, the synthesized AFRNBs@PtNPs showed consistency and well‐dispersion, respectively. Additionally, PtNPs on the AFRNBs were further observed by high‐resolution TEM (HRTEM) and showed an average lattice spacing of 0.226 nm, which was consistent with the (1 1 1) facet (Figure 1E). According to the X‐ray diffraction (XRD) pattern, 2θ of as‐prepared AFRNBs@PtNPs were located in 39.76°, 46.24°, 67.45°, 81.29°, and 85.72°, which were attributed to (111), (200), (220), (311), and (222) (JCPDS No.04–0802),^[^ ^7^ ^]^ respectively (Figure S1, Supporting Information), indicating the successful synthesis of PtNPs. These inconspicuous peaks demonstrated that the synthesized PtNPs were too small, which was consistent with the TEM image in Figure 1F. The isolated PtNPs could be more clearly observed, and the average size was about (1.57 ± 0.26) nm under HADDF mode. Moreover, the energy dispersive spectroscopy (EDS) mapping of AFRNBs@PtNPs was measured, and C, N, O, and Pt elements were all well‐distributed, demonstrating the Pt element was uniformly synthesized on the AFRNBs (Figure 1G), which was kept consistence with the X‐ray photoelectron spectroscopy (XPS) (Figure S2; Supporting Information). As shown in Figure 1H, peaks at ≈400.50 eV of AFRNBs@PtNPs and ≈401.40 eV of AFRNBs could be assigned to C═N,^[^ ^8^ ^]^ and at ≈399.24 eV of AFRNBs@PtNPs and at ≈399.40 eV of AFRNBs were because of C–N.^[^ ^9^ ^]^ Besides, the peak of N 1s of AFRNBs@PtNPs moved to the direction of high binding energy compared with AFRNBs, indicating the loss of electrons from N atoms. Besides, peaks at ≈531.18 eV of AFRNBs and at ≈531.39 eV of AFRNBs@PtNPs indicated the existence of ─OH.^[^ ^10^ ^]^ Binding energies at ≈532.91 eV of AFRNBs, and ≈532.70 and ≈534.15 eV of AFRNBs@PtNPs were generated by C─O.^[^ ^11^ ^]^ The negative binding energy shift of O 1s of AFRNBs@PtNPs demonstrated that O could receive electrons (Figure 1I). Furthermore, the positive binding energy shift of Pt 4f of AFRNBs@PtNPs indicated the loss of electrons of Pt atoms, demonstrating the possible interfacial electron transfer (N→Pt→O). Additionally, ≈71.29 and ≈75.10 eV, and ≈72.79 and ≈76.18 eV at the Pt 4f spectra of AFRNBs@PtNPs could be attributed to Pt^0^ and Pt^2+^ species, respectively (Figure 1J). Moreover, the Fourier transform infrared (FT‐IR) spectrum revealed that the peaks of AFRNBs@PtNPs were similar to the prepared AFRNBs, indicating the stability of supports in the synthesis of PtNPs (Figure S3; Supporting Information). Among them, C–H stretching, C–N stretching/N–H bending, and C═C stretching of aromatic rings of AFRNBs@PtNPs could be found at ≈1440, ≈1511, and ≈1621 cm^−1^, respectively.^[^ ^6^, ^12^ ^]^ Besides, the same synthesized process without AFRNBs was carried out, and the uneven PtNPs were formed, indicating AFRNBs were crucial for the synthesis of PtNPs (Figure S4; Supporting Information).

### 2.2 POD‐Like Activity of AFRNBs@PtNPs

**Figure 2:**
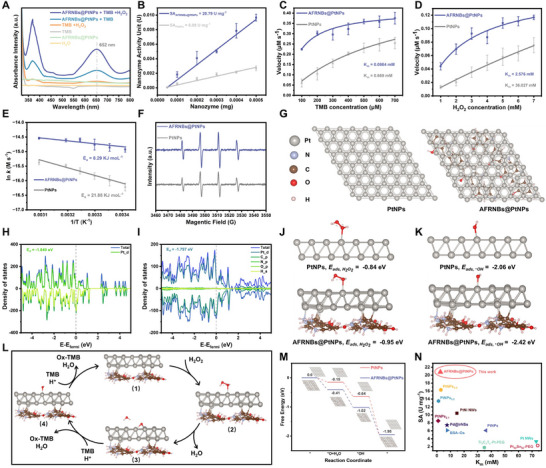
A) UV–vis spectra of AFRNBs@PtNPs‐catalyzed chromogenic reaction of TMB, B) SA of AFRNBs@PtNPs and PtNPs, data are expressed as the mean ± standard deviation (SD), with *n* = 3 independent samples, C) steady‐state kinetics concerning TMB, data are expressed as the mean ± SD, with *n* = 3 independent samples, D) steady‐state kinetics concerning H_2_O_2_, data are expressed as the mean ± SD, with *n* = 3 independent samples, E) the *E_a_* of AFRNBs@PtNPs and PtNPs, data are expressed as the mean ± SD, with *n* = 3 independent samples, F) ESR of AFRNBs@PtNPs and PtNPs, G) PtNPs and AFRNBs@PtNPs models, H) DOS of PtNPs, I) DOS of AFRNBs@PtNPs, J) $E_{ads,\;H_{2}O_{2}}$ of PtNPs and AFRNBs@PtNPs, K) *E_ads,_._OH_* of PtNPs and AFRNBs@PtNPs, L) POD‐like reaction process of AFRNBs@PtNPs, M) reaction pathways and free energies profiles for the POD‐like activity of PtNPs and AFRNBs@PtNPs, and N) scatter plot of AFRNBs@PtNPs and previously reported nanozymes.

To verify the POD‐like activity of AFRNBs@PtNPs, 3,3′,5,5′‐tetramethylbenzidine (TMB) was chosen as a catalytic substrate to study the catalytic property. As demonstrated in **Figure** **2**A, the classical chromogenic reaction of nanozyme‐catalyzed oxidation of TMB, which was measured by Ultraviolet visible (UV–vis) spectra. The characteristic peak of oxidized TMB (Ox‐TMB) indicated the POD‐like activity of AFRNBs@PtNPs. Additionally, the relationship between time and absorbance intensity at 652 nm was measured and indicated that the developed nanozyme‐catalyzed reaction was time‐dependent (Figure S5; Supporting Information), which was used to measure the nanozyme‐specific activity (SA) of AFRNBs@PtNPs.^[^ ^13^ ^]^ The SA of AFRNBs@PtNPs was 20.79 U mg^−1^, which was 3.4‐fold higher than that of PtNPs (Figure 2B). Additionally, the SA of developed AFRNBs@PtNPs was also superior to previous works, which was possibly because of AFRNBs supporting function (Table S1, Supporting Information). Based on this basis, the substrate affinity of AFRNBs@PtNPs and PtNPs was investigated through steady‐state kinetics testing according to Michaelis‐Menten equation.^[^ ^13^ ^]^ As illustrated in Figure 2C, the Michaelis‐Menten constant (K_m_) and maximum velocity (V_max_) of AFRNBs@PtNPs about TMB were 0.0984 mm and 0.444 µm s^−1^, respectively. While the K_m_ and V_max_ of PtNPs concerning TMB were calculated as 0.669 mm and 0.533 µm s^−1^, respectively. For H_2_O_2_, the K_m_ and V_max_ of AFRNBs@PtNPs were measured as 2.576 mm and 0.159 µm s^−1^, respectively, while the K_m_ and V_max_ of PtNPs were measured as 36.027 mm and 0.459 µm s^−1^, respectively (Figure 2D), indicating the higher substrate affinity. The *E_a_* was calculated by the Arrhenius Law (equation 1), where k, R, T, and C represented the reaction rate constant at temperature T, molar gas constant, absolute temperature, and constant of integration, respectively. The *E_a_* of AFRNBs@PtNPs was 8.29 KJ mol^−1^ while PtNPs was 21.88 KJ mol^−1^, demonstrating that AFRNBs could accelerate the POD‐like catalytic process (Figure 2E). From the perspective of intermediate generation^.^OH of AFRNBs@PtNPs and PtNPs was monitored by electron spin resonance (ESR), and AFRNBs@PtNPs exhibited higher^.^OH signal, beneficial in the efficient generation of catalytic intermediate (Figure 2F). Furthermore, the catalytic activity of developed AFRNBs@PtNPs was measured during 30 days at room temperature, demonstrating the role of AFRNBs support for nanozyme good stability (Figure S6; Supporting Information).

(1) $$lnk=\frac{-Ea}{RT}+C$$

To further reveal the AFRNBs on the nanozyme catalysis, DFT calculation was performed for PtNPs and AFRNBs@PtNPs (Figure 2G; Supporting Information). The density of states (DOS) for PtNPs and AFRNBs@PtNPs was presented in Figure 2H,I, respectively, exhibiting that the E_d_ relative to E_F_ of AFRNBs@PtNPs was upshifted from −1.849 to −1.797 eV compared with the one of PtNPs. The closer distance between E_d_ and E_F_ of AFRNBs@PtNPs could enhance the adsorption between the catalyst surface and H_2_O_2_ as well as oxygen intermediates, promoting the activation of H_2_O_2_. The absorption energy of H_2_O_2_ ($E_{ads,\;H_{2}O_{2}}$) was calculated as −0.95 and −0.84 eV for AFRNBs@PtNPs and PtNPs, respectively, demonstrating AFRNBs@PtNPs possessed a stronger adsorption for H_2_O_2_ over PtNPs (Figure 2J). Besides, the optimal value of absorption energy of ·OH radical (*E*_ads,·OH_), an effective descriptor for POD‐like activity for the highest POD‐like catalysis, was calculated as −2.6 eV.^[^ ^5^ ^]^ The *E*_ads,·OH_ was calculated as −2.06 and −2.42 eV for PtNPs and AFRNBs@PtNPs, respectively, exhibiting AFRNBs@PtNPs possessed the closest *E*_ads,·OH_ to the optimal value over PtNPs (Figure 2K), which was kept consistence with the ESR result. The electron transfer from PtNPs to AFRNBs could be the reason for the difference for $E_{ads,\;H_{2}O_{2}}$ and *E*_ads,·OH_. To further confirm this hypothesis, the reaction energy profile for POD‐like activity of PtNPs and AFRNBs@PtNPs was conducted. As shown in Figures 2L,M, the free energy of RDS (*H_2_O_2_ →*O+H_2_O) of AFRNBs@PtNPs and PtNPs were −0.41 and −0.15 eV, respectively, suggesting H_2_O_2_ could more easily react on the surface of AFRNBs@PtNPs than PtNPs. Finally, the possible electron transfer pathway of POD‐like catalysis was proposed (Scheme 1B): (1). Electron was transferred from PtNPs to AFRNBs through interfacial electron transfer (N→Pt→O); (2). PtNPs with electron deficiency exhibiting partially positive charge (δ+) could absorb the lone pair electrons in H_2_O_2_ and TMB to increase the interaction between nanozyme and substrates; (3). Electrons were transferred from the electron‐rich area to PtNPs and then to H_2_O_2_ to generate O* and H_2_O; (4). The absorbed TMB could be oxidized and lose electrons to O* to form *OH; (5). Electrons could be transferred from TMB to *OH and nanozymes to form H_2_O; (6). The accepted electron in PtNPs was then transferred to AFRNBs and recovered the initial state. The K_m_ about H_2_O_2_ and SA of the developed AFRNBs@PtNPs and previously reported nanozymes (Table S1, Supporting Information) were plotted in Figure 2N, indicating our prepared nanozyme has a good catalytic property.

### 2.3 AFRNBs@PtNPs‐catalyzed Multisignal Responses

**Figure 3:**
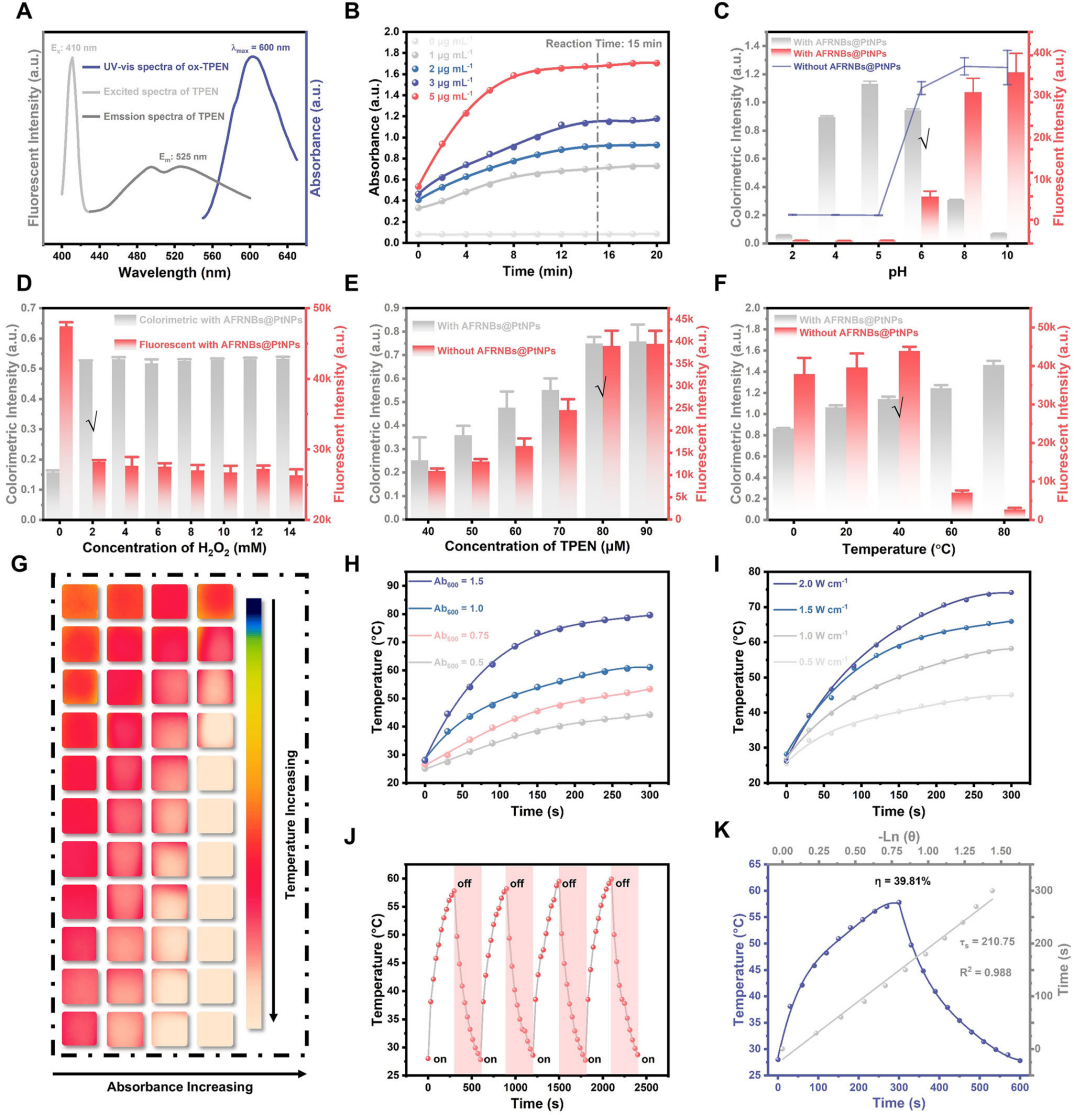
A) Multisignal responses of AFRNBs@PtNPs‐catalyzed AIEgens system, UV–vis spectra of ox‐TPEN and excited and emission spectra of TPEN, B) reaction time, C) the effect of pH, the blue line was the fluorescence intensity, data are expressed as the mean ± SD, with *n* = 3 independent samples, D) concentration of H_2_O_2_, data are expressed as the mean ± SD, with *n* = 3 independent samples, E) concentration of TPEN, data are expressed as the mean ± SD, with *n* = 3 independent samples, F) reaction temperature, data are expressed as the mean ± SD, with *n* = 3 independent samples, G) different absorbance of ox‐TPEN under 808 nm laser, H) the heating curve of ox‐TPEN with different absorbance, I) the heating curve of ox‐TPEN under different laser power, J) the heating and cooling circulate curve, and K) the photothermal conversion efficiency.

To construct a cross‐validated signal, TPEN AIEgens were used as the catalytic substrates of AFRNBs@PtNPs, and the catalytic products possessed triple signal responses, including colorimetric mode (CM), fluorescent mode (FM), and photothermal mode (PM).^[^ ^14^ ^]^ The triple signal responses depended on the lost electrons of TPEN to form positively charged oxidized TPEN (ox‐TPEN). First, the visual change could occur that TPEN was catalyzed by AFRNBs@PtNPs, which presented the change from colorless to bluish‐green, showing the absorbance peak at 600 nm in the UV–vis spectra. Meanwhile, the unreacted TPEN in the system could be excited by 410 nm to exhibit the 525 nm fluorescent emission (**Figure** **3**A). The nanozyme‐catalyzed system could rapidly reach the equilibrium time within 15 min, which guaranteed the requirement of rapid detection (Figure 3B). To optimize the multisignal response system, CM and FM were used as elements to study the reaction condition. As illustrated in Figure 3C–F, the pH, H_2_O_2_ concentration, TPEN concentration, and reaction temperature were optimized, respectively, which showed the optimal signal responses at pH = 6, H_2_O_2_ concentration = 2 mm, TPEN concentration = 80 µm, and reaction temperature = 40 °C, respectively. To investigate the photothermal property of ox‐TPEN, ox‐TPEN with different absorbances was irradiated by 808 nm laser, and their photothermal property depended on the absorbance (Figure 3G,H). Moreover, ox‐TPEN was irradiated by 808 nm laser with different powers, which indicated their photothermal property was also power‐dependent (Figure 3I). To study the stability of the catalytic products, ox‐TPEN was irradiated by 808 nm laser through four heating and cooling processes, which exhibited a similar highest temperature after four circulations, indicating their good stability of photothermal property (Figure 3J). To further evaluate the photothermal property of the nanozyme‐catalyzed system, the photothermal conversion efficiency (η) of ox‐TPEN was calculated as high as 39.81% according to the Equations S1 and S2 (Supporting Information; Figure 3K), indicating the good photothermal response of the nanozyme‐catalyzed system. Therefore, the AFRNBs@PtNPs nanozyme‐catalyzed AIEgens system enables triple‐signal output with bidirectional complementarity, demonstrating significant potential for multichannel sensing, such as sensor arrays and immunoassays.

### 2.4 Multisignal Responses‐Based Nanozyme Immunoassay

**Figure 4:**
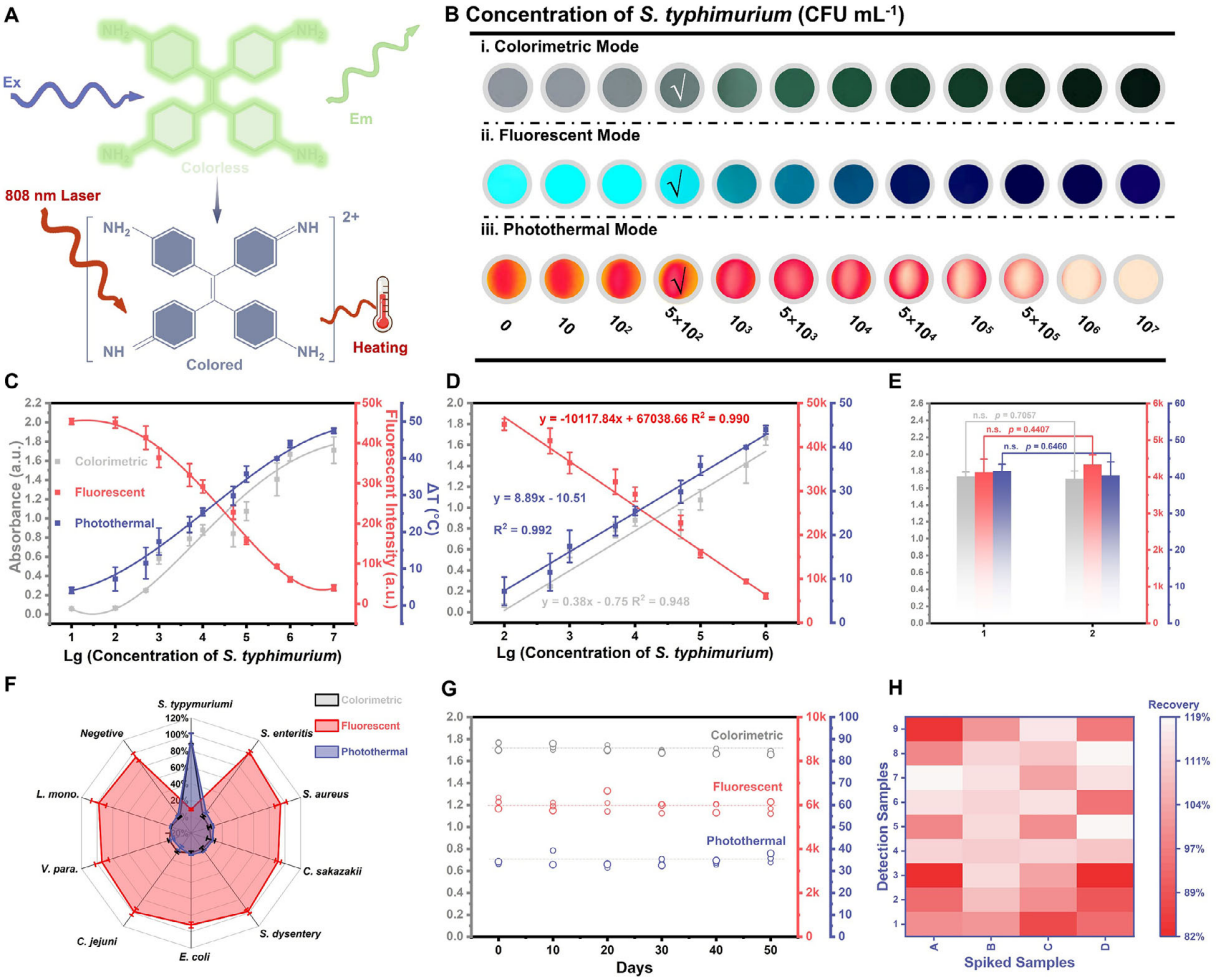
A) AFRNBs@PtNPs‐catalyzed TPEN for multisignal immunosensing, multisignal responses, B) image of multimodal immunoassay, C) multisignal value and *S. typhimurium* concentration, data are expressed as the mean ± SD, with *n* = 3 independent samples, and all y‐axes’ titles/units consistent with this, D) linear curve of the proposed immunoassay, data are expressed as the mean ± SD, with *n* = 3 independent samples, E) repeatability of the proposed method, data are expressed as the mean ± SD, with *n* = 3 independent samples, F) specificity of the developed immunoassay, data are expressed as the mean ± SD, with *n* = 3 independent samples, the Student's *t*‐test was utilized to assess the difference between the means of the two groups, and n.s. represents no significance (*p* > 0.05), G) stability of the proposed method, data are expressed as the single measurement value in 3 independent repeated experiments, and H) detection of *S. typhimurium* in real sample, A‐B represented 10^3^, 5 × 10^3^, 10^4^, and 5 × 10^4^ CFU mL^−1^, respectively, 1–3, 4–6, and 7–9 represented colorimetric, fluorescent, and photothermal, respectively, data are expressed as the single measurement value in 3 independent repeated experiment.

Based on the multisignal from AFRNBs@PtNPs‐catalyzed AIEgens, a multimodal immunoassay based on the classical sandwich method for the detection of *S. typhimurium* was constructed (**Figure** **4**A), whose detection condition was optimized in Figures S7 and S8(Supporting Information). As illustrated in Figure 4B, with the increase of *S. typhimurium* concentration, the CM, FM, and PM showed distinctive responses and could detect *S. typhimurium* at 500 CFU mL^−1^. As shown in Figure 4C, the *S. typhimurium* concentration presented a positive correlation with the signal responses in CM and PM and a negative correlation with the signal responses in FM, indicating the cross‐validated ability for biosensing. Afterward, the relationship between *S. typhimurium* and signal responses was scattered, and the linear curve was: i) y = 0.38x − 0.75 in the linear range of 10^2^‐10^6^ CFU mL^−1^ for CM (R^2^ = 0.948), whose limit of detection (LOD) was 220 CFU mL^−1^ (LOD was calculated by the average of blank + 3SD), ii) y = −10117.84x + 67038.66 in the linear range of 10^2^–10^6^ CFU mL^−1^ for FM (R^2^ = 0.990), whose LOD was 204 CFU mL^−1^ (LOD was calculated by the average of blank ‐ 3SD), iii) y = 8.89x − 10.51 in the linear range of 10^2^–10^6^ CFU mL^−1^ for PM (R^2^ = 0.992), whose LOD was 299 CFU mL^−1^ (LOD was calculated by the average of blank + 3SD), respectively (Figure 4D). Additionally, the linear curve of the conventional ELISA using HRP was: y = 0.27x – 0.45 in the linear range of 5 × 10^3^–10^7^ CFU mL^−1^ (R^2^ = 0.958), whose LOD was 1637 CFU mL^−1^ (LOD was calculated by the average of blank + 3SD) (Figures S9 and S10, Supporting Information), indicating the developed method could improve the detection sensitivity by one order of magnitude. Compared with previous works, the proposed immunoassay possessed a higher detection sensitivity and wider detection range (Table S2, Supporting Information). Besides, the relative standard deviation (RSD) in detecting different batches was 3.96%, 7.12%, and 6.47% for CM, FM, and PM, respectively, demonstrating the good repeatability of the developed method (Figure 4E). Afterward, *S. enteritis*, *S. aureus*, *C. sakazakii*, *S. dysentery*, *E. coli*, *C. jejuni*, *V. parahemolyticus*, *L. monocytogenes*, and *S. typhimurium* at 10^6^ CFU mL^−1^ were tested by the developed method to evaluate its specificity. The non‐targeted pathogens could be washed from the microwells by using *S. enteritis* as an example (Figure S11, Supporting Information). Only *S. typhimurium* could be detected as a positive result, which showed the specificity of detecting *S. typhimurium* (Figure 4F). Moreover, the stable multisignal during 50 days demonstrated the good stability of the developed method (Figure 4G). To evaluate the feasibility of fabricated immunoassay, *S. typhimurium* at the concentration of 10^3^, 5 × 10^3^, 10^4^, and 5 × 10^4^ CFU mL^−1^ spiked whole milk were tested by the developed immunoassay, whose recovery was ranging from 81.98% to 118.03%, and the RSD was below 16.90%, demonstrating the potential in solving pathogens monitoring in real‐world matrices (Figure 4H). It is expected that high‐performance nanozymes can be integrated with redox‐tunable AIEgens to achieve universal analyte detection through antibody (Ab) replacement.

### 2.5 Machine Learning‐Assisted Multisignal Biosensing and the SHAP Interpretable Frame Analysis

**Figure 5:**
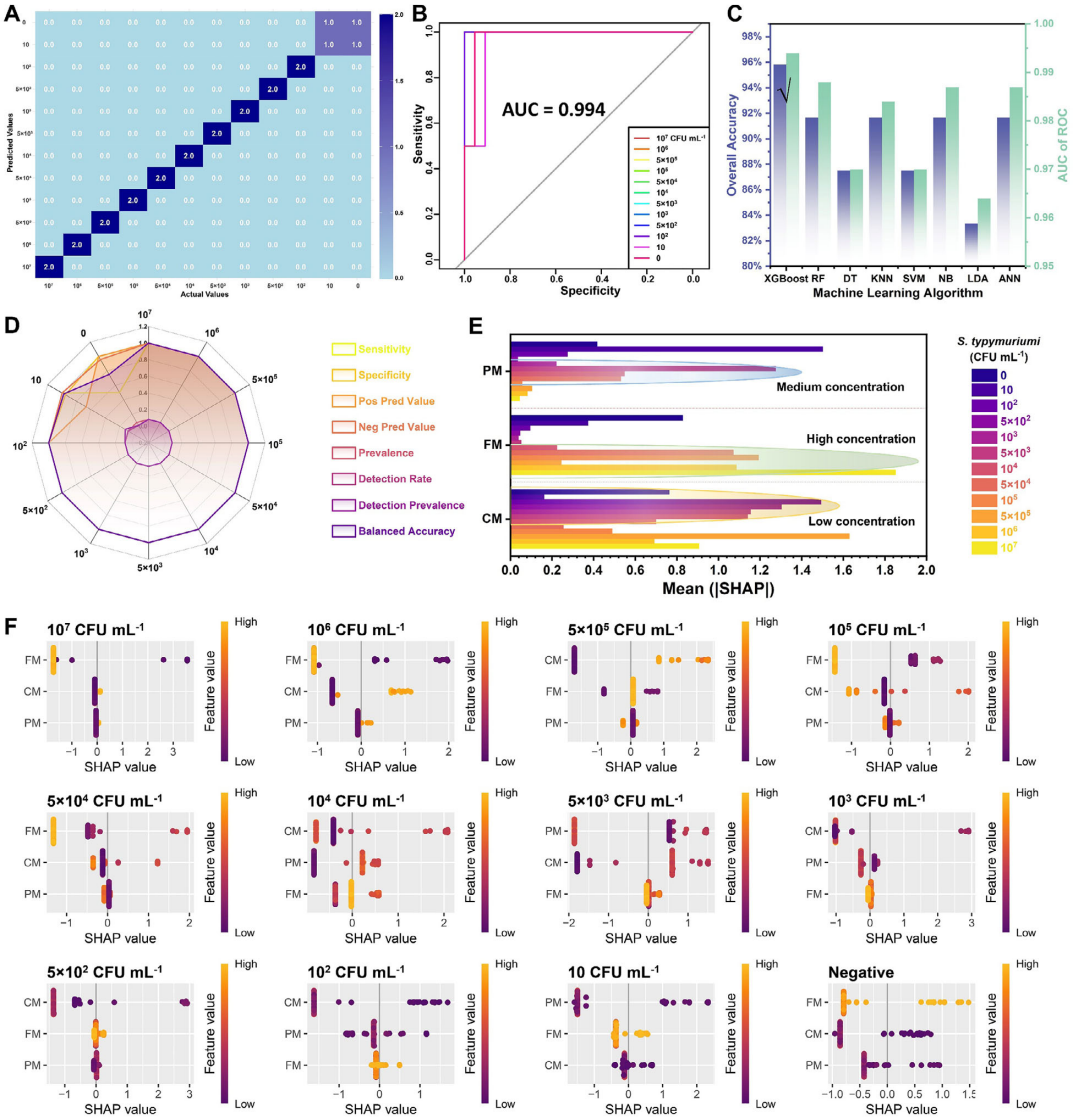
A) Machine learning algorithm‐assisted multisignal synergetic biosensor and the interpretable SHAP‐driven feature optimization in biosensing, confusion matrix of XGBoost algorithm, B) ROC curve of XGBoost algorithm, C) plot of overall accuracy and AUC, D) evaluation of XGBoost model, E) bar chart of the mean (|SHAP|) for multisignal, and F) SHAP value for the different concentrations of *S. typhimurium*.

XGBoost algorithm was used to fuse the multisignal of the nanozyme‐based biosensor to synergize and cross‐validate the triple signal for the improvement of their analytical performance. After dividing the dataset into 70% training set and 30% test set, the XGBoost algorithm was used to train and test the three‐channel signal dataset, and the overall accuracy of the model reached 95.83%. When the *S. typhimurium* concentration was more than 100 CFU mL^−1^, all positive samples could be accurately identified (**Figure** **5**A). Receiver operating characteristic (ROC) curve analysis showed that the average area under the curve (AUC) was 0.994, and the AUC reached 1.0 when the bacterial concentration was ≥100 CFU mL^−1^ (Figure 5B). Compared with the single‐signal detection mode, the multi‐signal collaborative strategy increased the detection sensitivity by five times, and the detection accuracy rate for positive samples ≥100 CFU mL^−1^ reaches 100%, proving that the multi‐signal fusion method is significantly superior to the single‐mode biosensing in terms of accuracy and sensitivity. Besides, XGBoost algorithm in dealing with the triple signal set stood up in accuracy and sensitivity than the traditional machine learning algorithms, which possessed different data dealing models, including random forest (RF) (AUC = 0.988, completely identified >500 CFU mL^−1^), decision tree (DT) (AUC = 0.970, completely identified >500 CFU mL^−1^), k nearest neighbor (KNN) (AUC = 0.984, completely identified >500 CFU mL^−1^), support vector machine (SVM) (AUC = 0.970, completely identified>500 CFU mL^−1^), naive bayes (NB) (AUC = 0.987, completely identified>500 CFU mL^−1^), linear discriminant analysis (LDA) (AUC = 0.964, completely identified>500 CFU mL^−1^), and artificial neural network (ANN) (AUC = 0.987, completely identified>500 CFU mL^−1^) algorithms (Figure 5C). As illustrated in Figure 5D, sensitivity, specificity, pos pred value, neg pred value, prevalence, detection rate, detection prevalence, and balanced accuracy showed a good detection performance for *S. typhimurium*. To vitrify the black‐box model of XGBoost algorithm‐assisted nanozyme‐based multisignal biosensors, SHAP framework, a method based on game theory, was used to quantify the contribution difference of each biosensing signal according to the Equation (2), where *F*, *S*, *j*, *f(S)*, and $f\left(S\cup \left\{j\right\}\right)$ represented a set of all features, a subset of a certain feature, the current assessed feature, predictive value of model trained only by *S*, and predictive value of model trained with feature *j*, respectively, which could measure the marginal contribution of a feature and take the weighted average under all possible feature combinations.

(2) $$\phi_{j}=\sum_{S\subseteq F\setminus \left\{j\right\}}\frac{|S|!\left(|F|-|S|-1\right)!}{|F|!}\left[f\left(S\cup \left\{j\right\}\right)-f\left(S\right)\right]$$

In the non‐linear XGBoost algorithm, the influence of multisignal could be changed by the interaction effect, and the SHAP value could well explain the dynamic change. As illustrated in Figure 5E, the calculated SHAP value of multisignal in the global interpretation of XGBoost model was represented, which exhibited the triple signals on the prediction results of *S. typhimurium* detection. The |SHAP| of triple signals in each *S. typhimurium* concentration was ranked, showing the ranking and relative influence of triple signals on 12 kinds of *S. typhimurium* categories predicted by the XGBoost model. For example, FM was the most influential signal (1.851) and PM was the least influential signal (0.044) in the detection of *S. typhimurium* at 10^7^ CFU mL^−1^, demonstrating the difference of the triple signal in the contribution to biosensing. Besides, the contribution of CM was largest at low concentration, PM at medium concentration, and FM at high concentration, showing the concentration‐dependent signal contribution, which could be a feedback on the single‐mode biosensing to enhance the detection reliability. This difference in triple signals could be attributed to the signal generation mechanisms of the developed biosensors. The colorimetric and photothermal signals derive from oxidized AIEgens (with the latter requiring η), while fluorescence originates from non‐oxidized AIEgens. As demonstrated in Figure 5F, the real SHAP value not only explained the importance of features, but also showed the actual relationship between features and prediction results, further demonstrating the different signals possessed the importance difference in detecting different *S. typhimurium*. The feature dependence was shown in Figures S12–S14(Supporting Information), the nonlinear relationship between the SHAP value of FM, CM, or PM and the feature value demonstrated that the prediction result of this feature not only depended on the value of the feature, but also may be influenced by other features. Single‐sample interpretation showed that the model could capture the complex interaction between signals and identify *S. typhimurium* categories by combining the contributions of different features (Figure S15; Supporting Information). In conclusion, the SHAP value could effectively explain the prediction behavior of the model, providing strong support for the interpretability of the XGBoost model.

## 3 Conclusion

In conclusion, we reported an electron transfer‐tailored E_d_ to boost nanozyme catalysis coupled with AIEgens for the ultrasensitive detection of pathogens through interpretable machine learning. The interfacial electron transfer between PtNPs and AFRNBs upshifts the Pt E_d_ relative to the E_F_ to optimize H_2_O_2_ adsorption energy, lowers the energy barrier of the RDS, and reduces *E_a_*, resulting in a 3.4‐fold enhancement in POD‐like catalysis over Pt nanozymes. By integrating with AIEgens, cross‐validated anti‐interference signals were developed for immunoassay, achieving a record‐low LOD of 204 CFU mL^−1^ for *S. Typhimurium*, exceeding conventional immunoassays in sensitivity and accuracy. The XGBoost‐SHAP framework dynamically fused multimodal signals, improving the sensitivity by 5‐fold over single‐mode detection and delivering 100% diagnostic accuracy for positive samples. The SHAP further revealed the synergetic mechanism of multimodal signals, and found concentration‐dependent signal contributions and validated decision logic. This work not only established a new concept for the rational design of nanozymes, but also refined biosensing paradigms through machine learning‐powered signal intelligence, which transformed biosensors from passive transducers to active diagnosis, offering a blueprint for combating global health crises.

## 4 Experimental Section

### Synthesis of AFRNBs@PtNPs

AFRNBs@PtNPs were synthesized by the following method. First, AFRNBs were acquired by the previous work with some modification.^[^ ^6b^ ^]^ Briefly, 100 mg of m‐aminophenol was added to 30 mL of pure water containing 100 µL of ammonia and then uniformly stirred. Afterward, 100 µL of formaldehyde was added to the mixture and then kept stirring for 30 min at room temperature. Then, 100 mL of anhydrous ethanol was added to etch the formed sphere‐like AFR for 3 h at room temperature. Subsequently, the obtained AFRNBs were purified by centrifugation at 8000 rpm and 10 min. The prepared AFRNBs were resuspended in 30 mL pure water, and then 25 mg K_2_PtCl_4_ and 3 mL 200 mm ascorbic acid were added to the solution for 4 h at 80 °C. Finally, the synthesized AFRNBs@PtNPs were obtained by centrifugation at 8000 rpm and 10 min to remove the unreacted reagent. The prepared AFRNBs@PtNPs were stored at 4 °C for further use.

### Preparation of Capture Abs‐Modified Microplates

Briefly, 200 µL capture Abs were added to microplates and kept incubated at 4 °C overnight. And then, the detection microplates were washed with PBST (10 mm PBS and 0.5% Tween‐20) for three times. Afterward, the detection microplates were sealed by 2% BSA for 40 min at 37 °C. Subsequently, the detection microplates were washed with PBST for three times. Finally, the detection microplates were stored at 4 °C for further use as soon as possible.

### Preparation of AFRNBs@PtNPs‐Detection Abs Complex

Briefly, 10 µL detection Abs (1 mg mL^−1^) were added into 1 mL AFRNBs@PtNPs (1 mg mL^−1^) and then kept still for 2 h at 37 °C. Afterward, the complex was sealed by 2% BSA through incubation for 2 h at 37 °C. And then, the complex was washed with 10 mm PBS (pH 7.4) three times to remove the uncombined Abs. Finally, the prepared complex was stored at 4 °C for further use as soon as possible.

### Triple Responses‐Based Immunoassay

The proposed immunoassay was carried out by the following steps. Briefly, 200 µL *S. typhimurium* or food samples were added into microplates for 40 min at 37 °C. The microplate was washed with PBST for three times. After that, 200 µL AFRNBs@PtNPs‐detection Abs complex (300 µg mL^−1^) was added into microplates for 40 min at 37 °C. The microplate was washed with PBST for six times. Subsequently, 200 µL reaction system containing 80 µm TPEN and 2 mm H_2_O_2_ in NaAc‐HAc buffer (pH 6.0) was added into microplates at 40 °C for 15 min. The absorbance, fluorescent intensity, and photothermal signal were measured by the above method.

### Statistical Analysis

Origin 2024 software, R 4.4.1, and GraphPad Prism 10.4.2 were utilized to draw figures and perform statistical analysis. Data in the figures were presented as the mean ± SD from three independent replicate experiments (*n* = 3). Multisignal generated by nanozyme‐based biosensor was analyzed and processed by a stretch of open R packages in R 4.4.1. Package “caret” was used to train the training set. XGBoost, RF, DT, KNN, SVM, NB, LDA, and ANN algorithms were realized by the package “xgboost,” “randomForest,” “rpart,” “class,” “e1071,” “e1071,” “MASS,” and “nnet,” respectively. ROC curve analysis was conducted by R 4.4.1. The Student's t‐test was utilized to assess the difference between the means of the two groups, and n.s. represents no significance (*p* > 0.05).

## Conflict of Interest

The authors declare no conflict of interest.

## Supporting information



Supporting Informartion

## Data Availability

The data that support the findings of this study are available from the corresponding author upon reasonable request.
