# Supplementary material for: Electron Transfer‐Tailored D‐Band Center to Boost Nanozyme Catalysis for Interpretable Machine Learning‐Empowered Intelligent Biosensing
Source: Adv Sci (Weinh). 2025 Aug 23;12(42):e05712. doi: 10.1002/advs.202505712 (PMC12622424; doi:10.1002/advs.202505712)
Supplement: Supplementary file 1 — Supporting Informartion [file ADVS-12-e05712-s001.docx]

***Supporting Information for***

# Electron Transfer-Tailored D-band Center to Boost Nanozyme Catalysis for Interpretable Machine Learning-Empowered Intelligent Biosensing

Yuechun Li,^[a]^ Chenxin Ji,^[a]^ Zhaowen Cui,^[a]^ Jianxing Feng,^[a]^ Liang Zhang,^[a]^ Sha Liu,^[b]^ Wentao Zhang,^[a]^ Yanwei Ji,^[a]^ Yizhong Shen,*^[b]^ and Jianlong Wang ^*[a]^

^a^ College of Food Science and Engineering, Northwest A&F University, 22 Xinong Road, Yangling 712100, Shaanxi, China.

^b^ School of Food & Biological Engineering, Hefei University of Technology, Hefei 230009, China

*Corresponding author.

E-mail: yzshen@hfut.edu.cn; wanglong79@nwsuaf.edu.cn

**Content**

[Materials and Reagents S3](#_Toc204772113)

[Culture of Bacteria S4](#_Toc204772114)

[Application in Real Samples S4](#_Toc204772115)

[DFT Calculation S4](#_Toc204772116)

[AFRNBs@PtNPs-catalyzed Triple Signal Responses S5](#_Toc204772117)

[Photothermal Conversion S6](#_Toc204772118)

[Figure S1 S7](#_Toc204772119)

[Figure S2 S8](#_Toc204772120)

[Figure S3 S9](#_Toc204772121)

[Figure S4 S10](#_Toc204772122)

[Figure S5 S11](#_Toc204772123)

[Figure S6 S12](#_Toc204772124)

[Figure S7 S13](#_Toc204772125)

[Figure S8 S14](#_Toc204772126)

[Figure S9 S15](#_Toc204772127)

[Figure S10 S16](#_Toc204772128)

[Figure S11 S17](#_Toc204772129)

[Figure S12 S18](#_Toc204772130)

[Figure S13 S19](#_Toc204772131)

[Figure S14 S20](#_Toc204772132)

[Figure S15 S21](#_Toc204772133)

[Table S1. S22](#_Toc204772134)

[Table S2 S23](#_Toc204772135)

[References S24](#_Toc204772136)

# Materials and Reagents

m-Aminophenol, formaldehyde, ammonia, potassium platinic chloride (K_2_PtCl_6_), ascorbic acid, 3,3',5,5'-tetramethylbenzidine (TMB), hydrogen peroxide (H_2_O_2_), and tetrakis(4-aminophenyl)ethene (TPEN) were purchased from Sigma-Aladdin (Shanghai, China). Paired sandwich anti-*S. typhimurium* monoclonal antibodies and horse radish peroxidase-labeled detection antibodies were prepared by our lab. Bovine serum albumin (BSA) was obtained from MP Biomedicals. *Salmonella typhimurium* (*S. typhimurium*) ATCC 43174, *Salmonella enteritidis* (*S. enteritidis*) ATCC 13076, *Cronobacter sakazakii* (*C. sakazakii*) CICC10899, *Vibrio parahemolyticus* (*V. parahaemolyticus*) ATCC178022, *Staphylococcus aureus* (*S. aureus*) ATCC29213, *Escherichia coli O157:H7* (*E. coli O157:H7*) ATCC 43889, *Listeria monocytogenes* (*L. monocytogenes*) CMCC54004, *Shigella dysentery* (*S. dysentery*) and *Campylobacter jejuni* (*C.* *jejuni*) were all preserved in our laboratory. Milk samples were purchased from a local supermarket in Yangling. Additionally, all solvents and other chemicals without special statement used in the work were analytical-reagent grades.

Transmission electron microscope (TEM) images were obtained by the HT7800 (Hitachi, Japan). Powder X-ray diffraction (XRD) pattern was measured by the powder diffractometer (Bruker D8 Advanced Diffractometer System, Germany) with a Cu Kα (1.5418 Å) source. X-ray photoelectron spectrometer (XPS) was employed an Axis Ultra DLD X-ray photoelectron spectrometer equipped with Al Kα X-ray as the excitation source (1486.6 eV). Fourier transform infrared spectroscopy (FT-IR) was studied by vertex70. Ultraviolet-visible (UV-vis) absorption spectra was measured by a double-beam spectrometer (MAPADA, China). The fluorescent spectra measurements were used a LS-55 spectrometer (PerkinElmer, Britain). ESR spectra was measured by Bruke EMX PLUS. Well-wash 4 Mk 2 microplate strip washer (Thermo Electron Corporation), ELISA plates were measured by Spark microplate reader (Tecan Austria GmbH, Austria), 96-well high-binding ELISA strip plates (Corning Costar Co., Ltd., Germany) were used in the process of ELISA.

# Culture of Bacteria

Bacteria were inoculated onto solid medium plates by plate streaking method, and incubated at 37 °C overnight. Afterwards, pure bacterial colonies with good growth state were selected on the plate, and cultured overnight in a liquid medium of a 250 mL conical flask by shaking at 37 °C overnight (200 rpm). The obtained bacteria were washed with 10 mM PBS, and the residual medium was removed by centrifugation at 8,000 rpm for 10 min.

# Application in Real Samples

To evaluate the feasibility of developed method, milk samples were chosen as real sample to be tested by our proposed method. Briefly, *S. typymurium*-uncontaminated milk was obtained in local supermarket in Yangling and confirmed by GB 4789.4-2024. Then, *S. typymurium* at 2×10^3^, 10^4^, 2×10^4^, and 10^5^ CFU mL^-1^ were spiked to prepare *S. typymurium*-contaminated milk and then diluted by 2-fold through 10 mM PBS. The test steps were kept consistence with our developed method.

# DFT Calculation

The Density Functional Theory (DFT) calculations were carried out by using the Vienna Ab-initio Simulation Package (VASP) ^1, 2^. The Perdew-Burke -Ernzerhof (PBE) functional under the generalized gradient approximation (GGA) method was employed to depict the exchange-correlation effects ^3, 4^. The projected augmented wave (PAW) method was utilized to describe the core-valence interactions ^5^. A plane-wave energy cutoff of 400 eV was adopted, and 1×1×1 Gamma-centered k-points were chosen for Brillouin zone integration. For structural optimization, the convergence criteria for energy and force were set at 1.0×10⁻⁴ eV and 0.05 eV Å⁻¹, respectively.

The adsorption energy (E_ads_) is defined as:

$$E_{ads}=E_{complex}-E_{substrate}-E_{adsorbate}$$

Here, “substrate” and “adsorbate” respectively denote the substrate surface and the adsorbate molecule. A lower adsorption energy indicates higher stability.

The Gibbs free energy changes (Δ*G*) of the reaction are calculated using the following formula:

$$\Delta G=\Delta E\text{+}\Delta ZPE-T\Delta S+\Delta G_{U}+\Delta G_{pH}$$

In this context, Δ*E* represents the electronic energy difference directly derived from DFT calculations, while ΔZPE stands for the zero-point energy difference. *T* is the room temperature, specifically 298.15 K, and *ΔS* denotes the entropy change. The term Δ*G*_U_ is defined as −e*U*, with *U* being the applied electrode potential. For Δ*G*_pH_, it is calculated as *k*_B_*T* × ln 10 × pH, where *kB* is the Boltzmann constant, and the pH value is set at 0.

# AFRNBs@PtNPs-catalyzed Triple Signal Responses

TPEN was chosen as the substrate of AFRNBs@PtNPs with POD-like activity for colorimetric/fluorescent/photothermal responses. Briefly, AFRNBs@PtNPs was added into the 200 μL reaction system containing 80 μM TPEN and 2 mM H_2_O_2_ in NaAc-HAc buffer (pH = 6.0) at 40 °C. After reacting for 15 min, the absorbance of the solution was measured at 600 nm and the fluorescent intensity was measured at the excited wavelength of 410 nm and emission wavelength of 525 nm. After catalysis, the solution could be measured by 808 nm laser to obtain the photothermal signal.

# Photothermal Conversion

Photothermal conversion efficiency (η) was measured by the equation S1&S2:

$$ƞ=\frac{hs\left( T_{max}-T_{surr} \right)-Q_{dis}}{I(1-{10}^{-A_{\lambda}})} (eq S1)$$

$$t=-\tau_{s}ln\theta=\frac{\sum_{i} m_{i}C_{p,i}}{hs}\ln\left( \frac{T-T_{surr}}{T_{max}-T_{surr}} \right)(eq S2)$$

In the provided equation, the variables are defined as follows: $h$ (convective heat transfer coefficient), $s$ (specific surface area of the container), $T_{max}$ (peak temperature of the sample solution), $T_{surr}$ (ambient temperature), *Q_dis_* (thermal energy variation in the blank reagent), *I* (incident laser power), and $A_{\lambda}$ (absorbance of the sample at 808 nm). Additionally, *m_i_* and *C_p,i_* represent the mass and specific heat capacity of the solvent (water), respectively. The system parameters η (thermal activation constant) and $\tau_{s}$ (relaxation time constant) are determined by analyzing the linear correlation between time *t* and *-*$ln\theta$, achieved through regression modeling.

# Figure S1


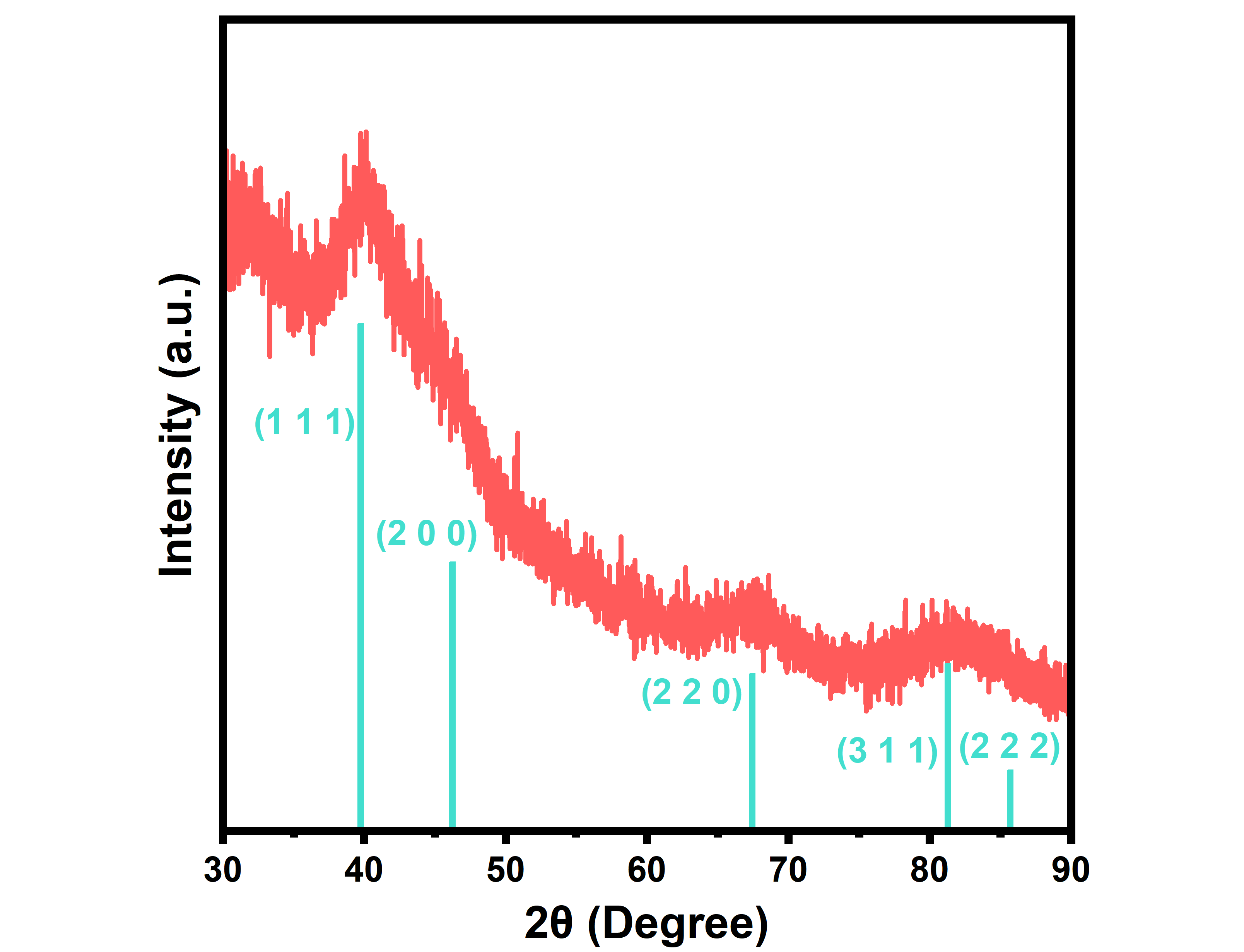
 **Figure S1.** XRD pattern of prepared AFRNBs@PtNPs.

# Figure S2


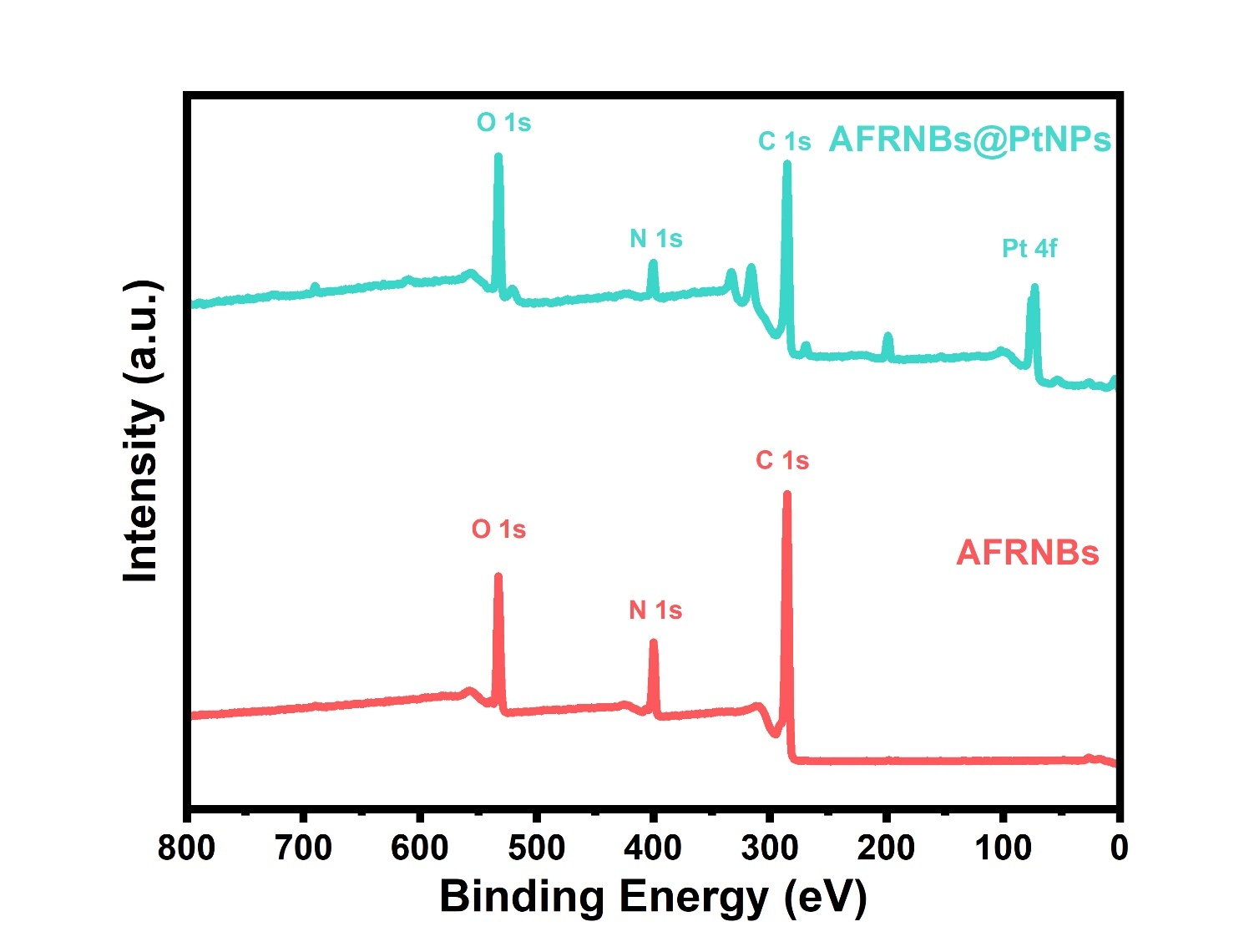
  **Figure S2.** XPS of prepared AFRNBs@PtNPs and AFRNBs.

# Figure S3


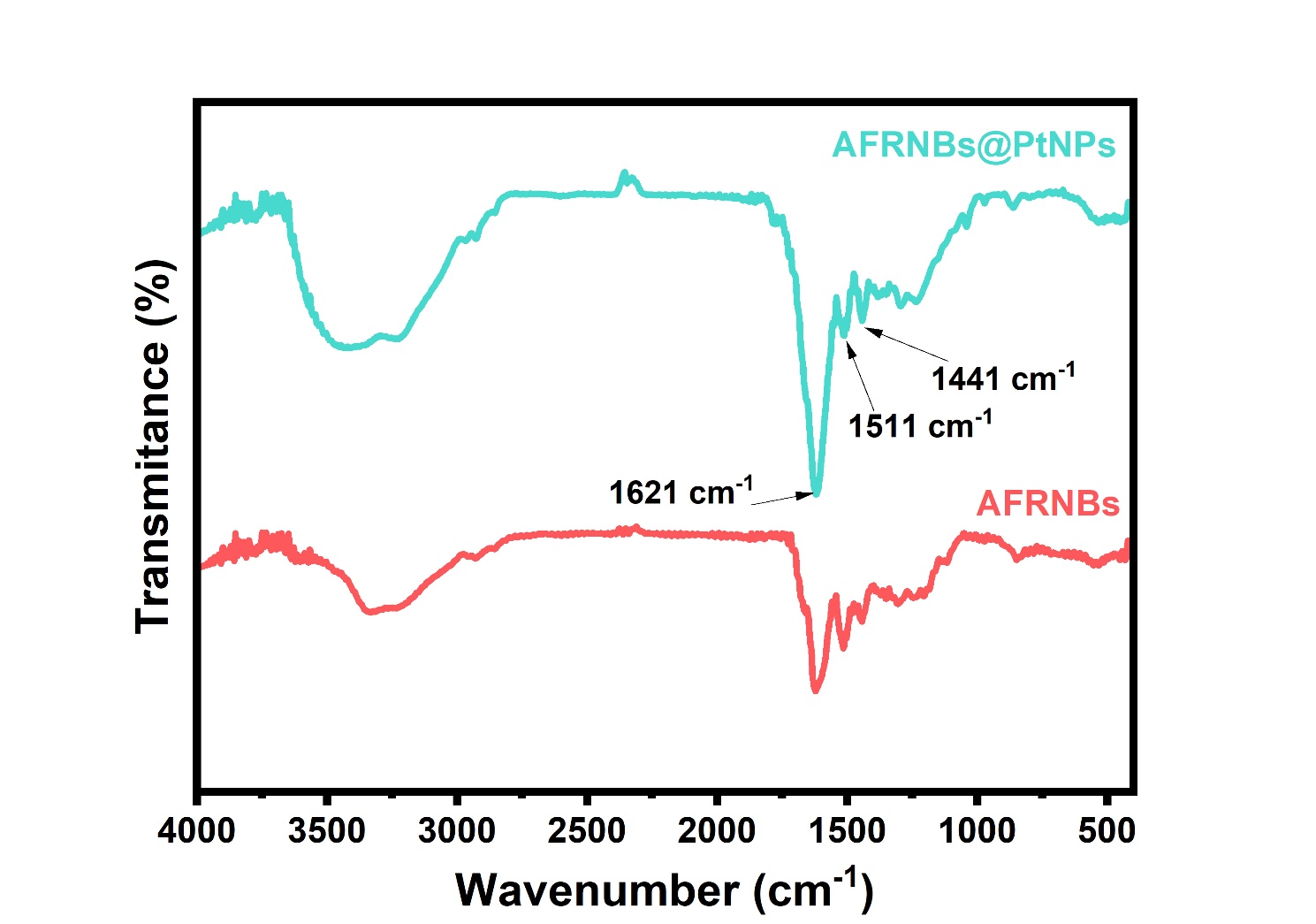
 **Figure S3.** FT-IR spectra of prepared AFRNBs@PtNPs and AFRNBs.

# Figure S4


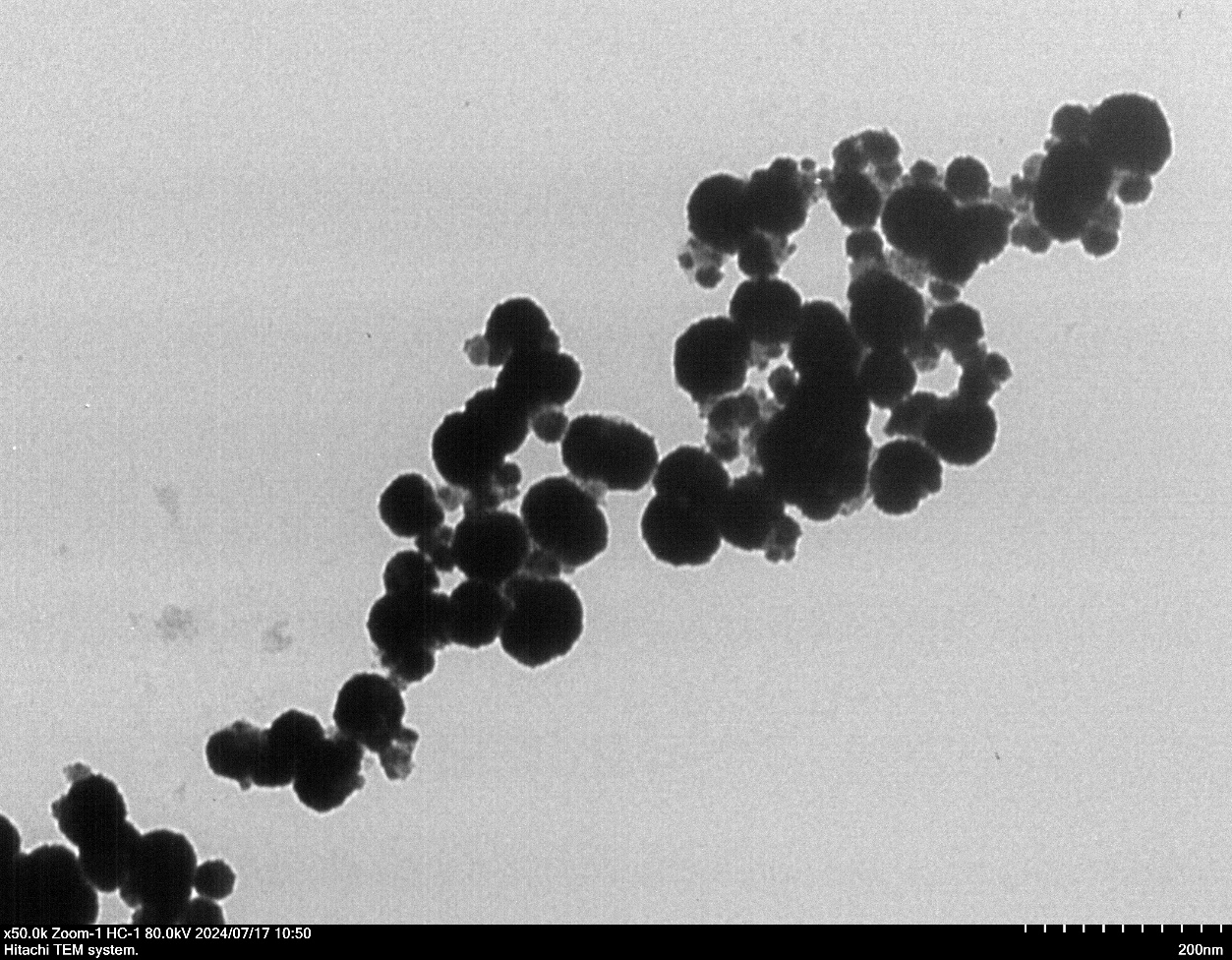


**Figure S4.** TEM image of prepared PtNPs without the participation of AFRNBs.

# Figure S5


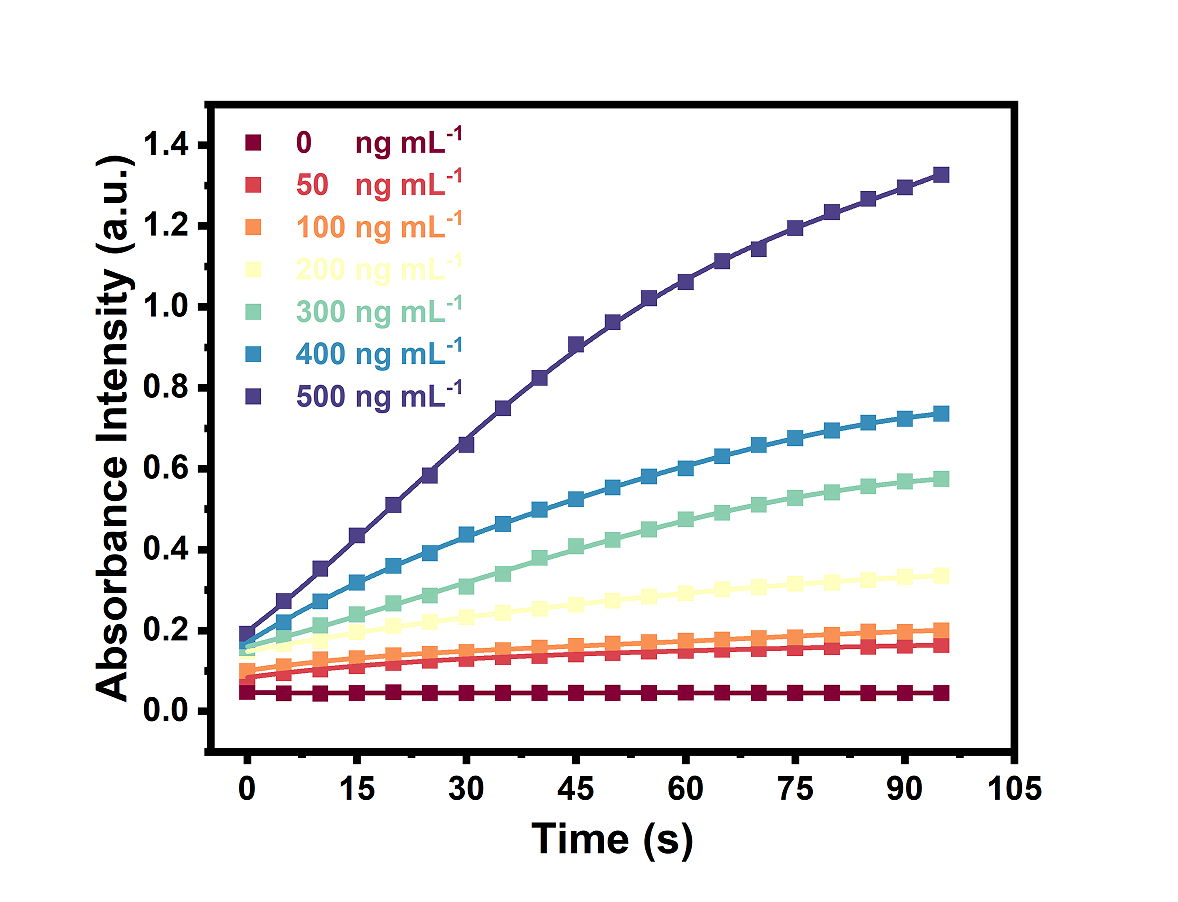


**Figure S5.** The relationship between reaction time of AFRNBs@PtNPs and absorbance intensity.

# Figure S6


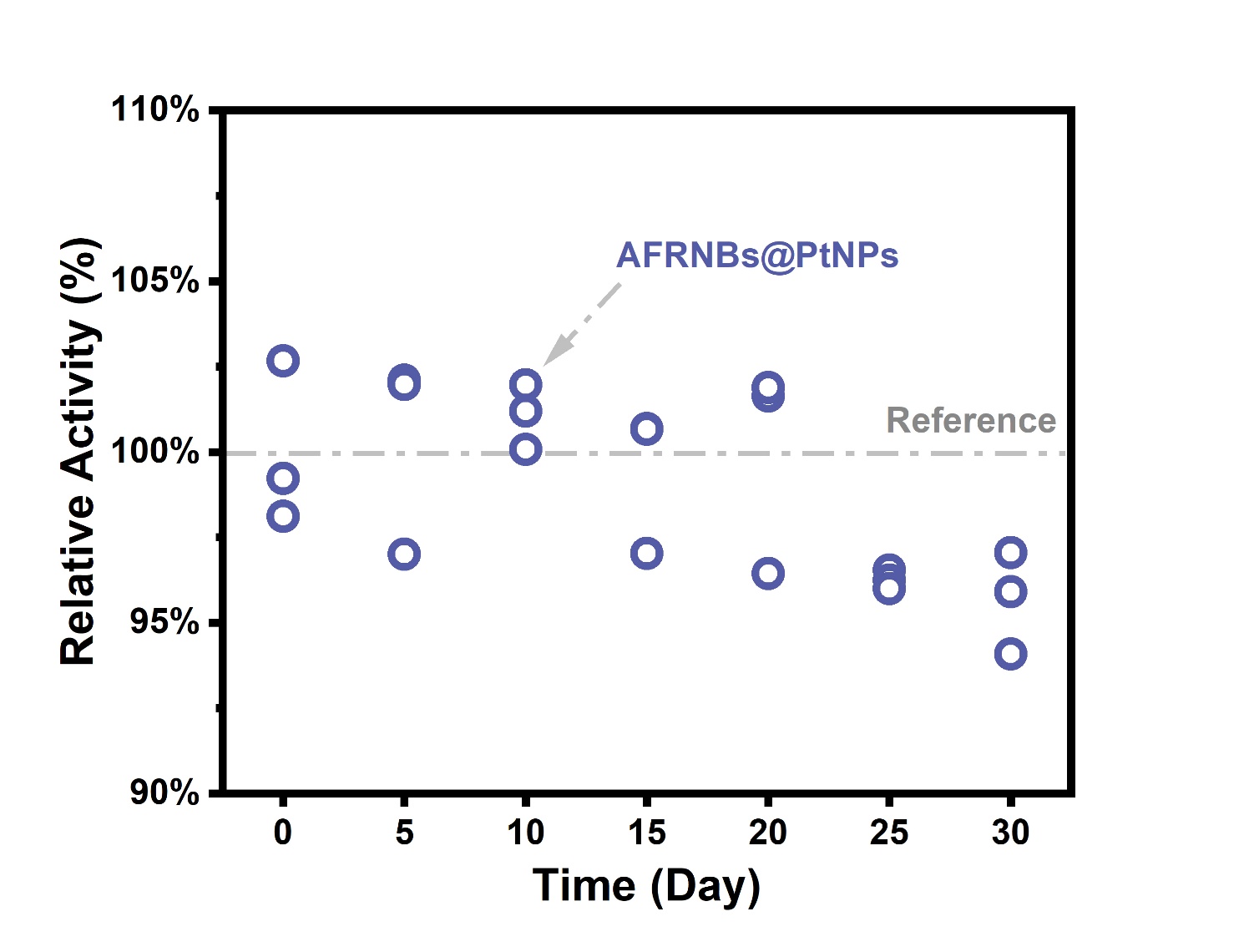


**Figure S6.** The POD-like stability of AFRNBs@PtNPs.

# Figure S7


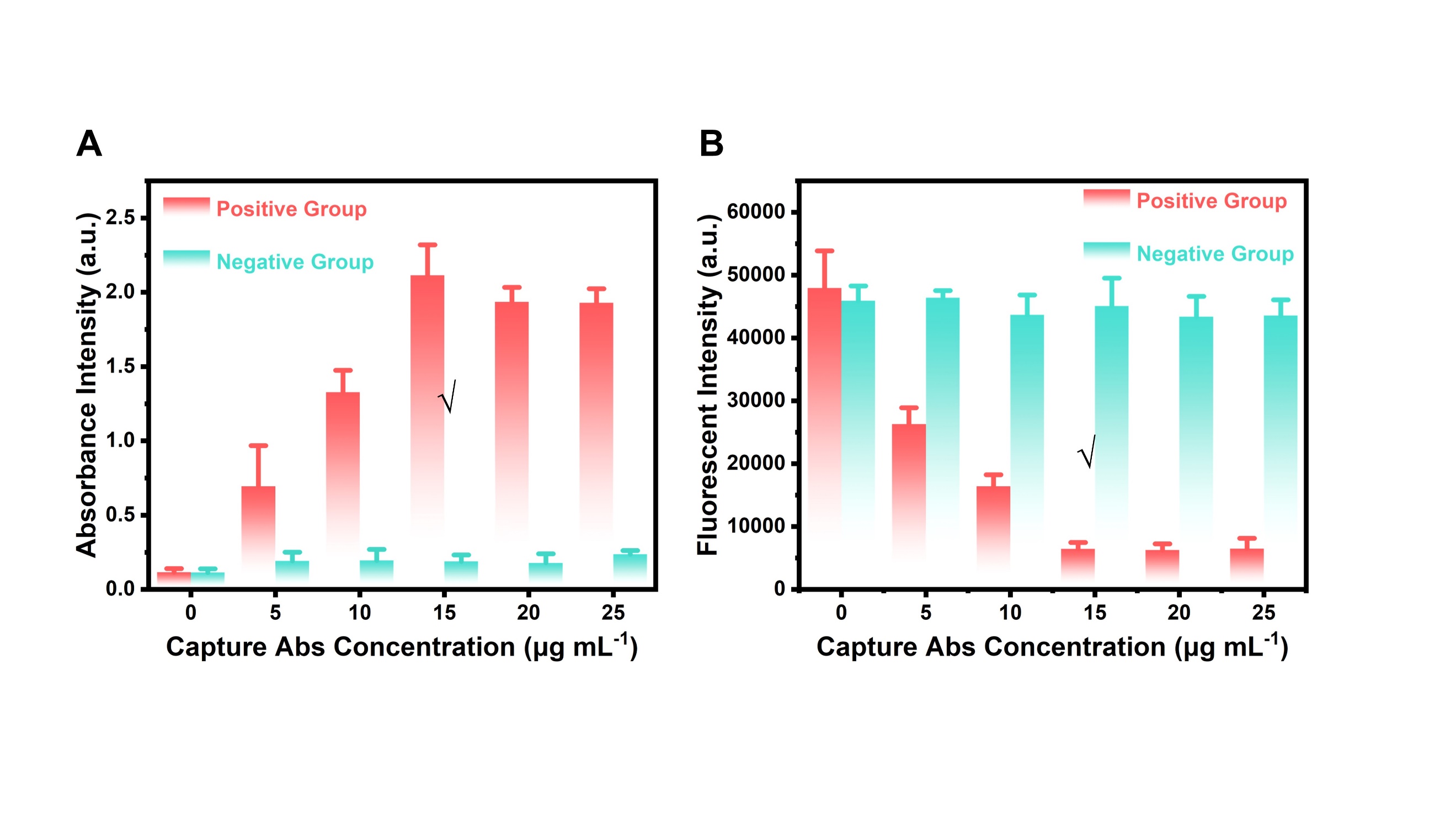


**Figure S7.** The optimization of capture Abs concentration for the developed ELISA. (A) Colorimetric mode. (B). Fluorescent mode.

# Figure S8


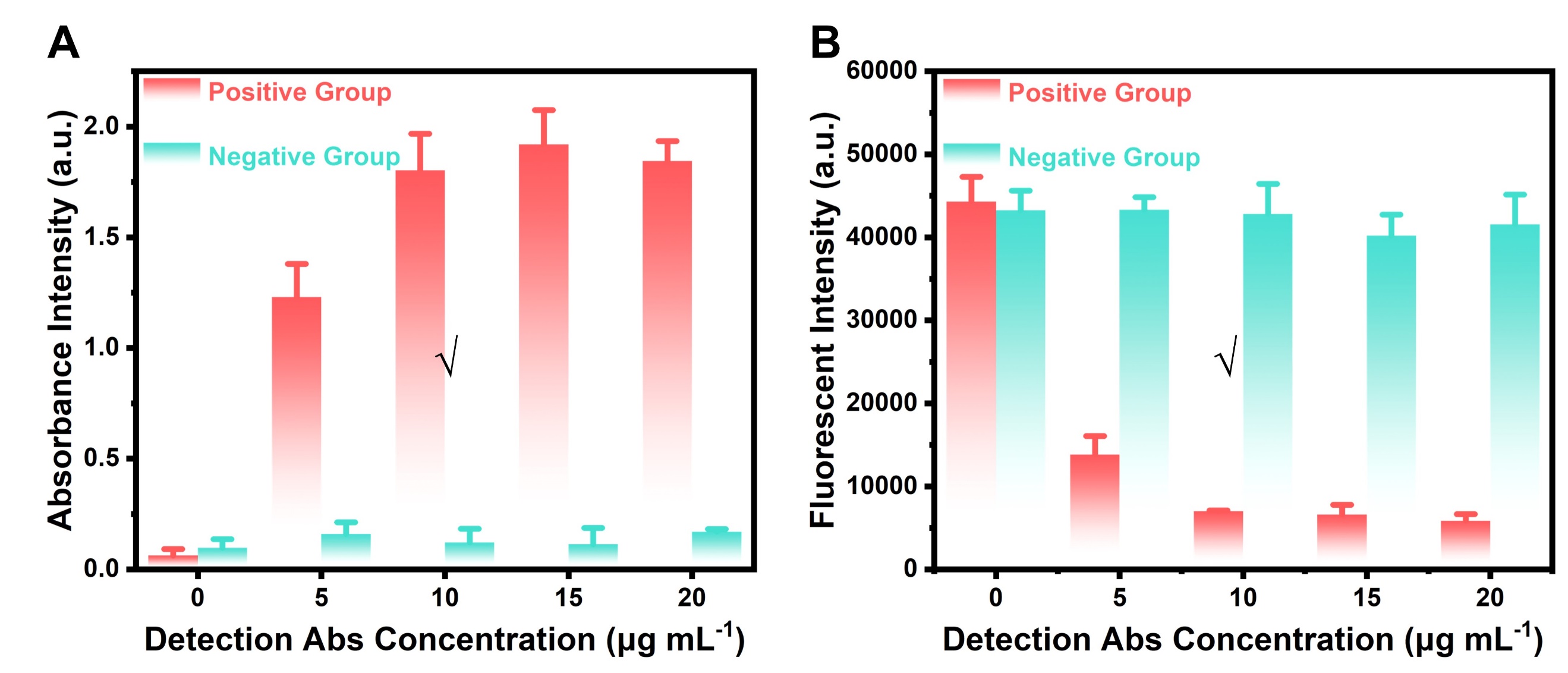


**Figure S8.** The optimization of detection Abs concentration for the developed ELISA. (A) Colorimetric mode. (B). Fluorescent mode.

# Figure S9


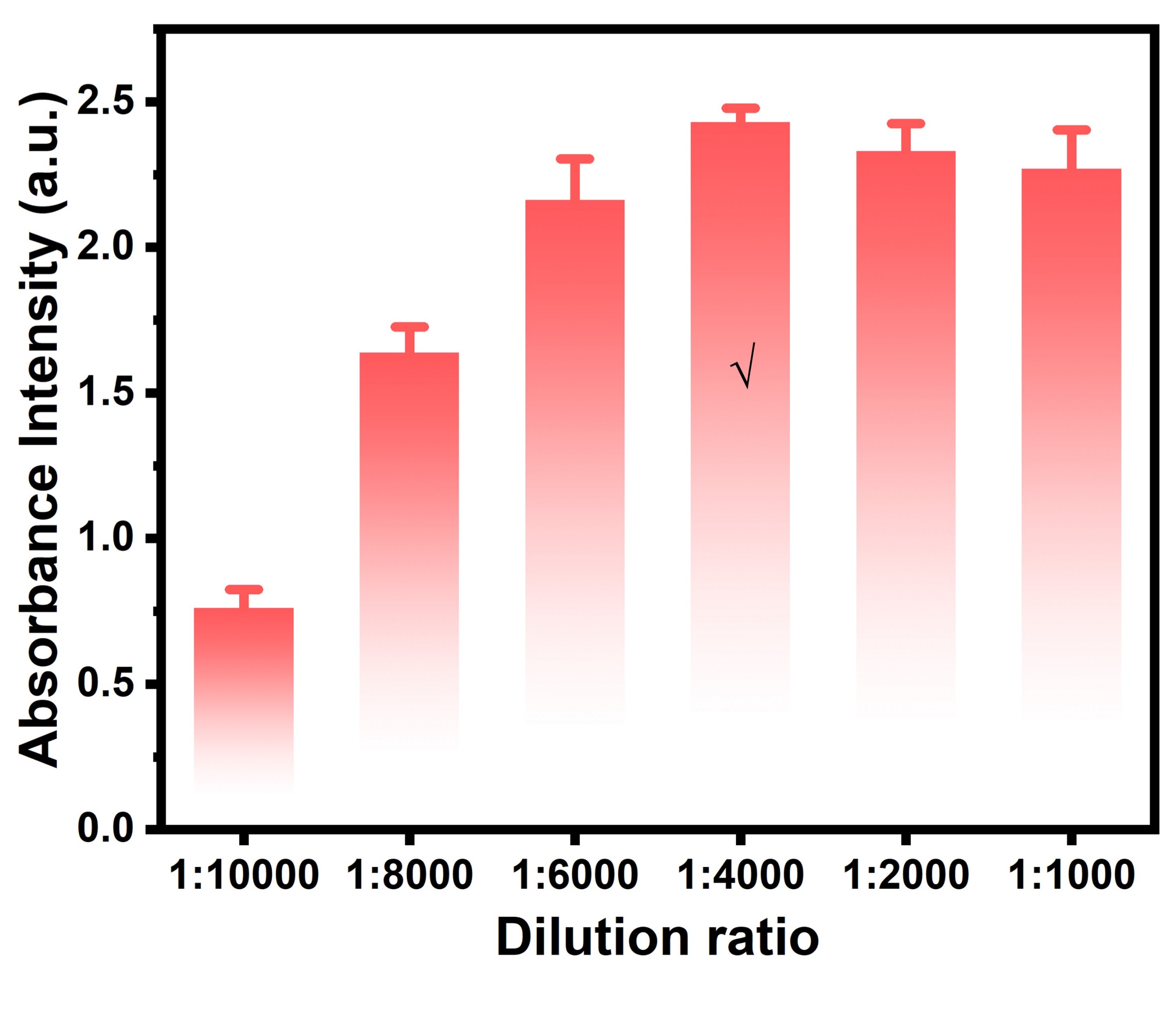


**Figure S9.** The optimization of HRP-detection Abs for the conventional ELISA.

# Figure S10


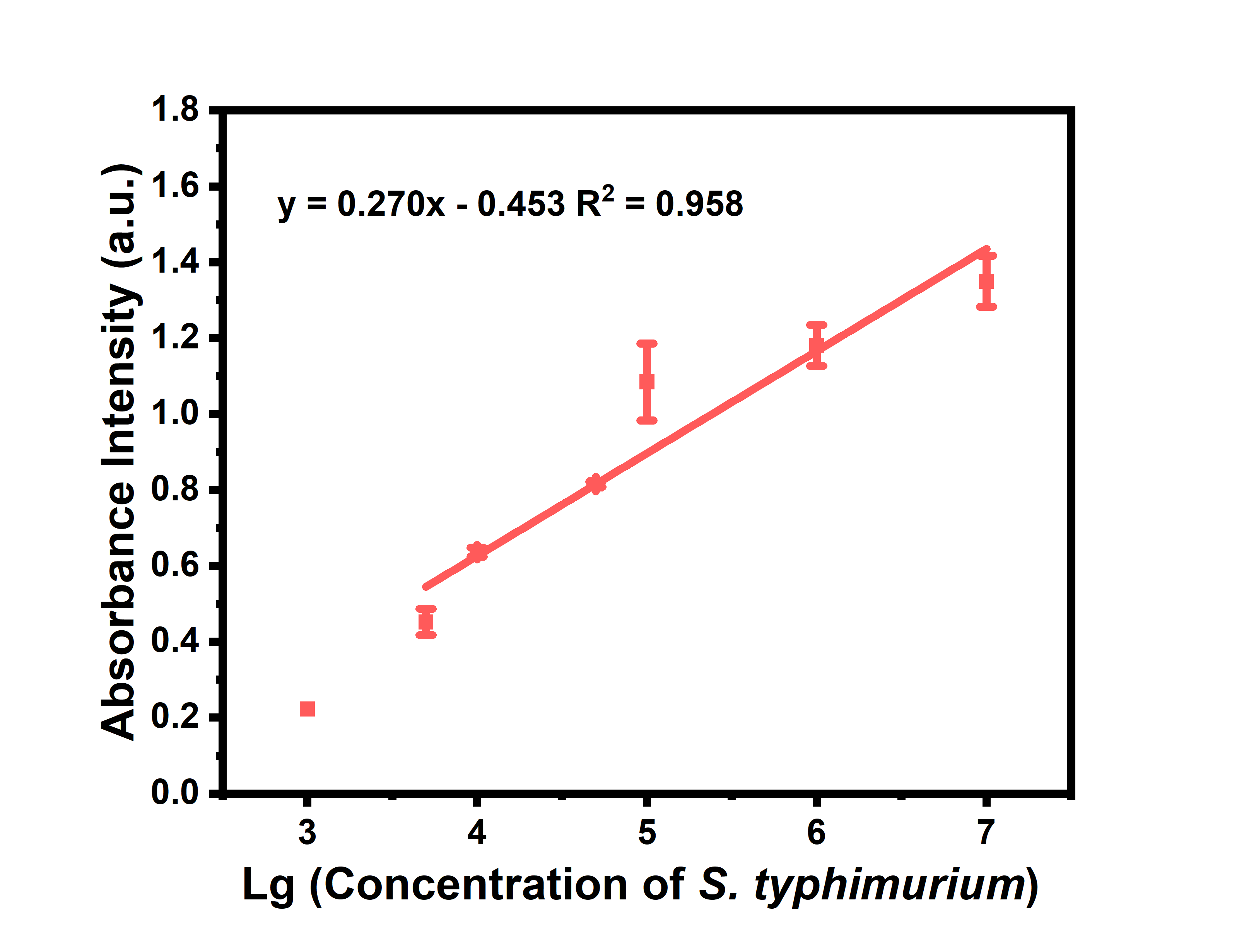


**Figure S10.** Detection of *S. typhimurium* by using conventional ELISA. All experiments were kept consistence with the developed nano-ELISA except for HRP-detection Abs.

# Figure S11


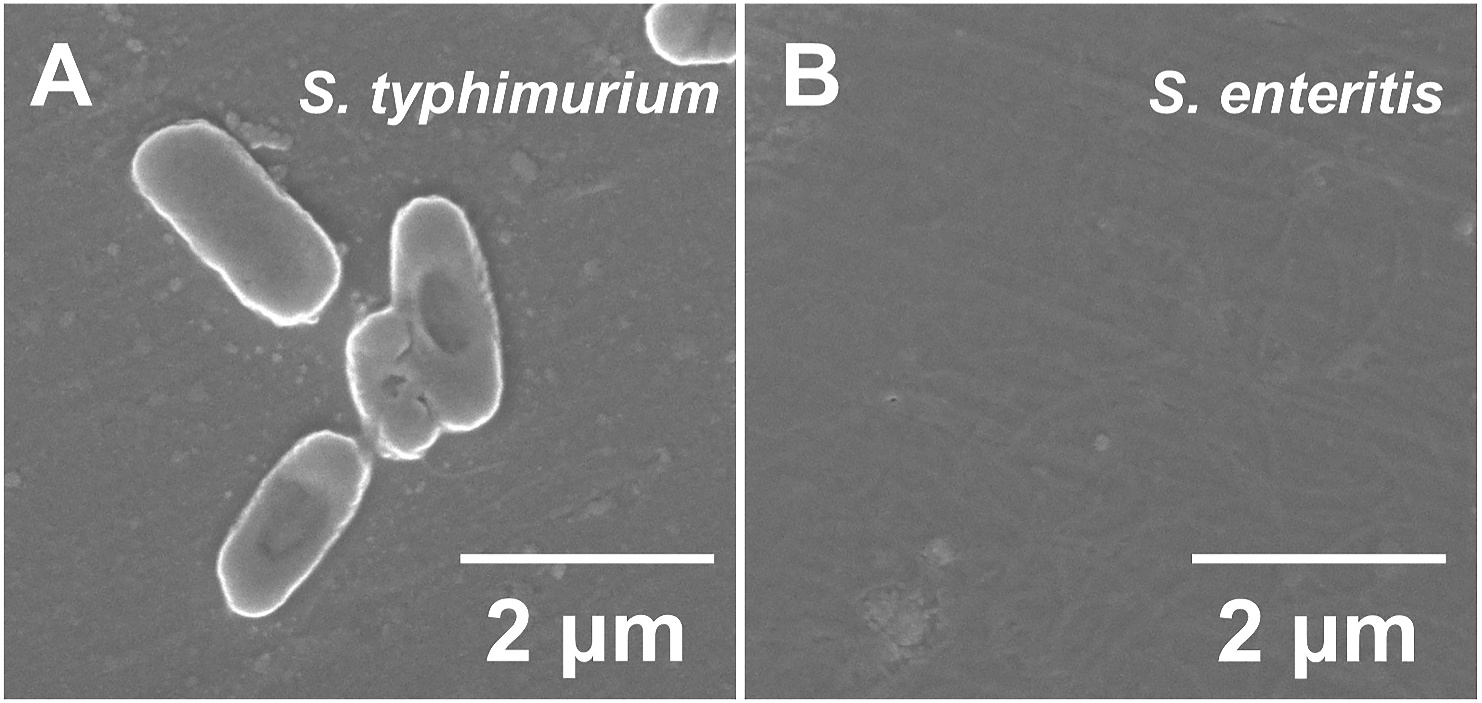


**Figure S11.** SEM images of detecting *S. typhimurium* and the non-target pathogens. (A). *S. typhimurium*, (B). Non-target pathogen using *S. enteritis* as an example.

# Figure S12


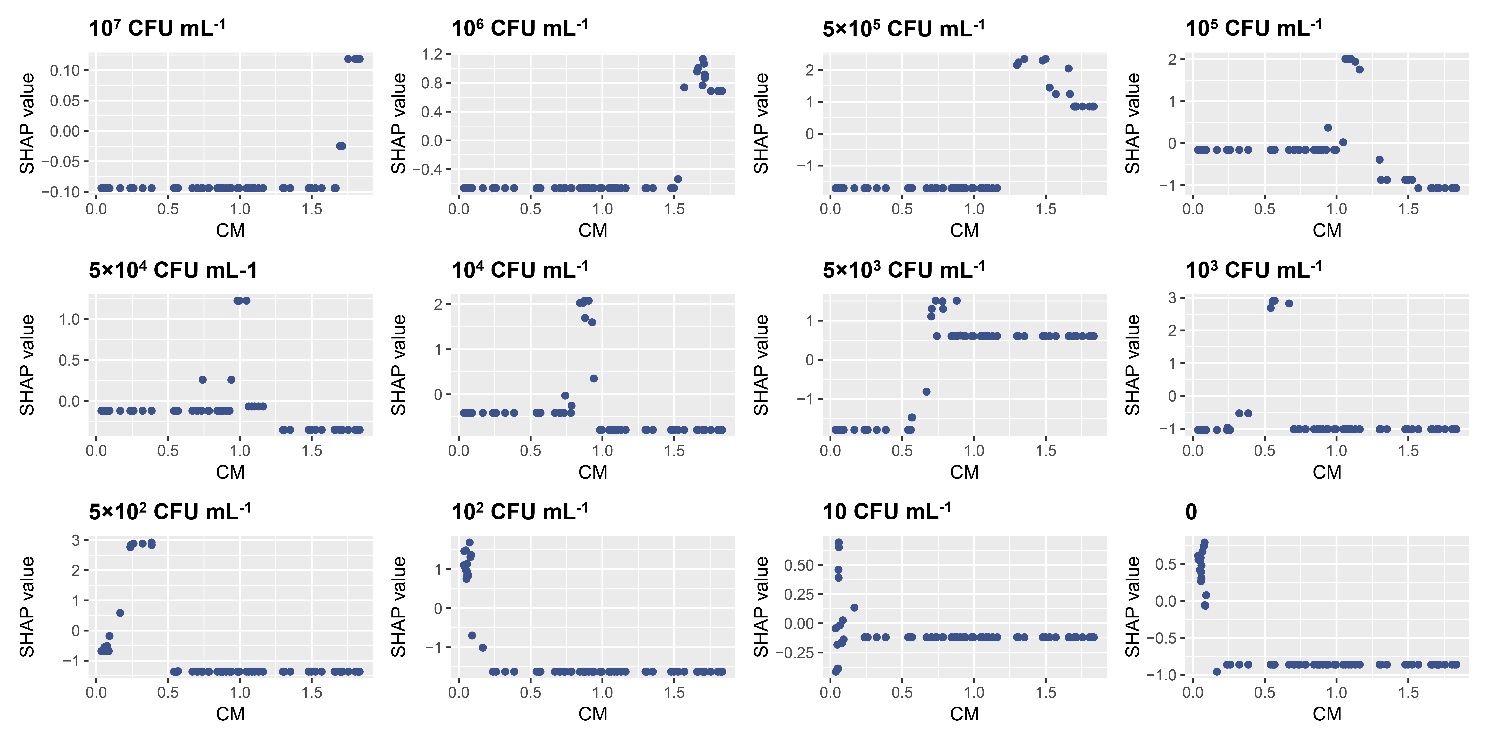
 **Figure S12.** The nonlinear relationship between SHAP value of CM and the feature value.

# Figure S13


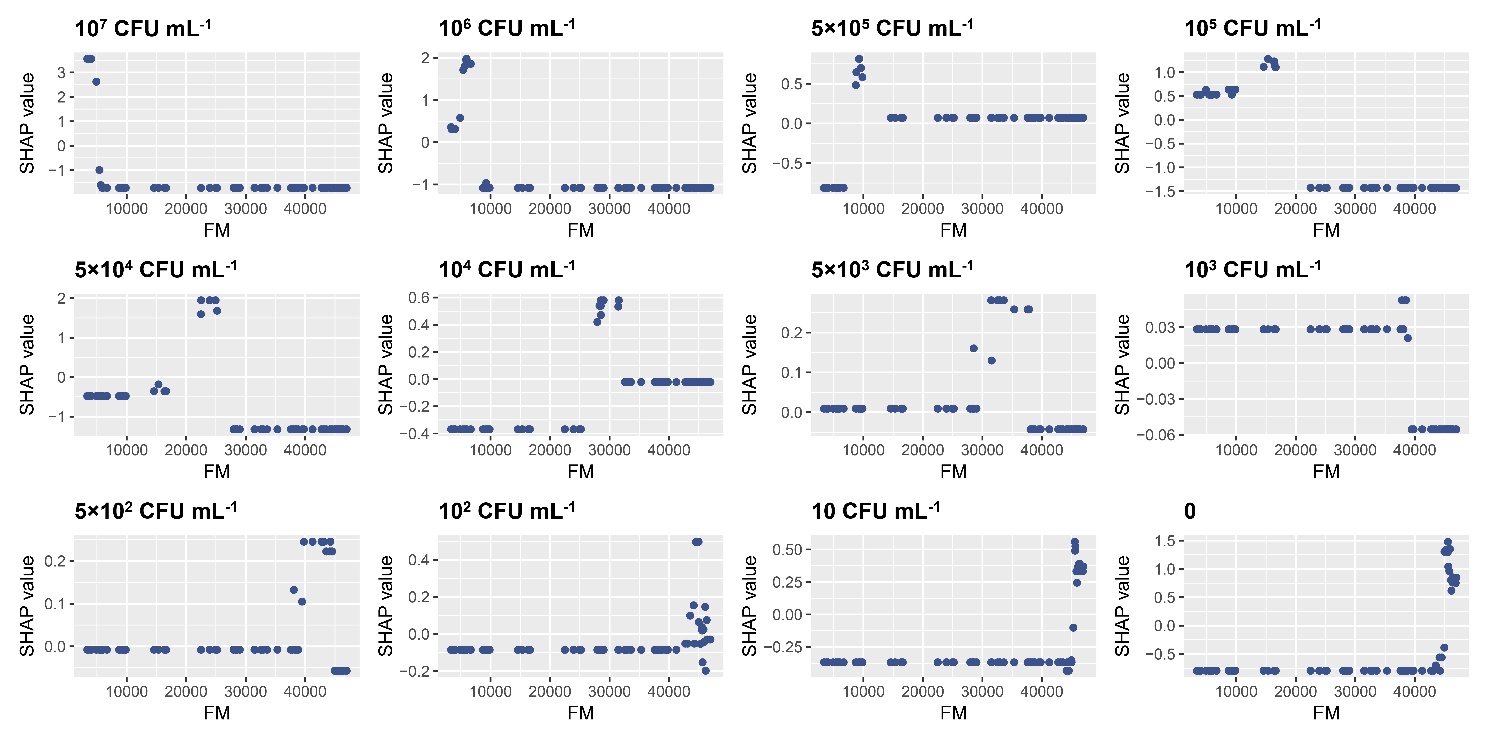
 **Figure S13.** The nonlinear relationship between SHAP value of FM and the feature value.

# Figure S14


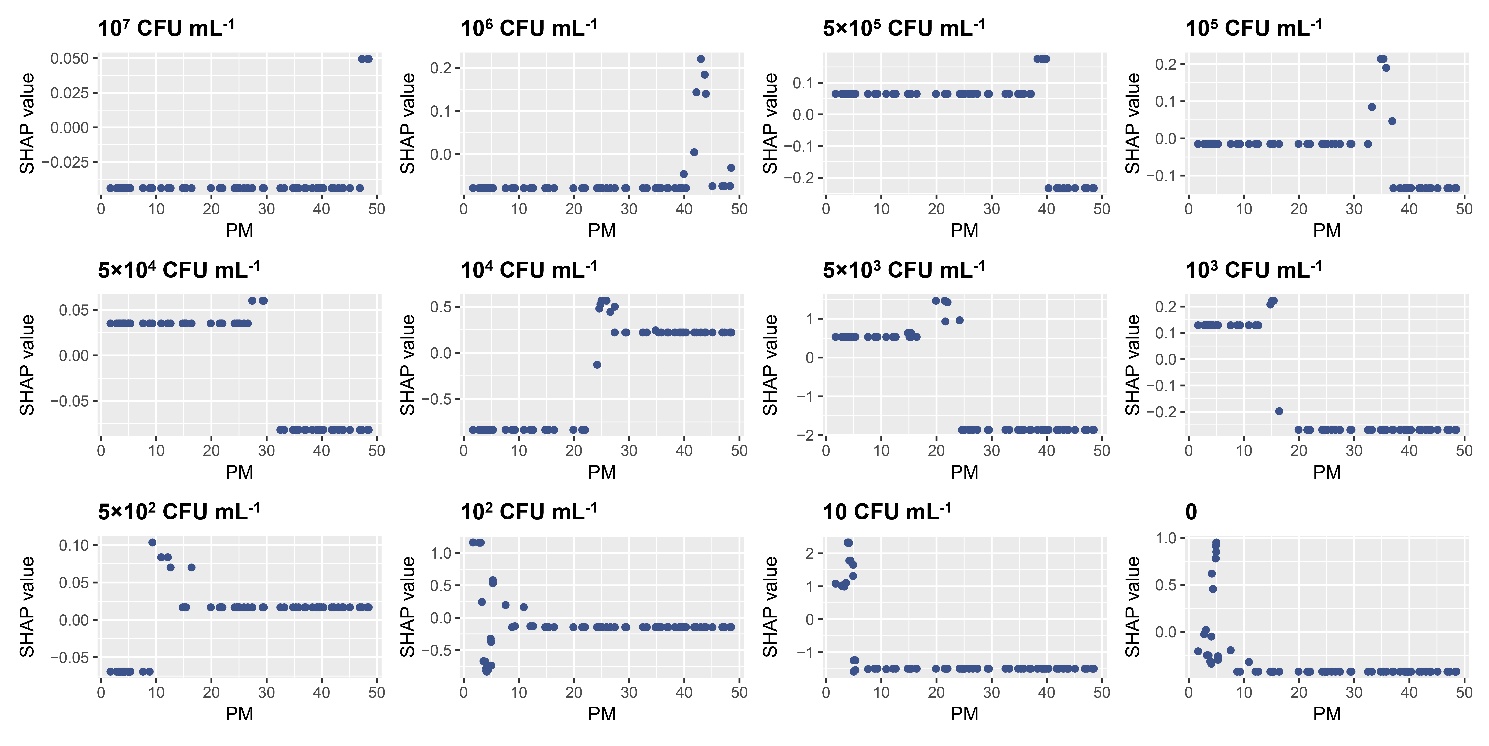
 **Figure S14.** The nonlinear relationship between SHAP value of PM and the feature value.

# Figure S15


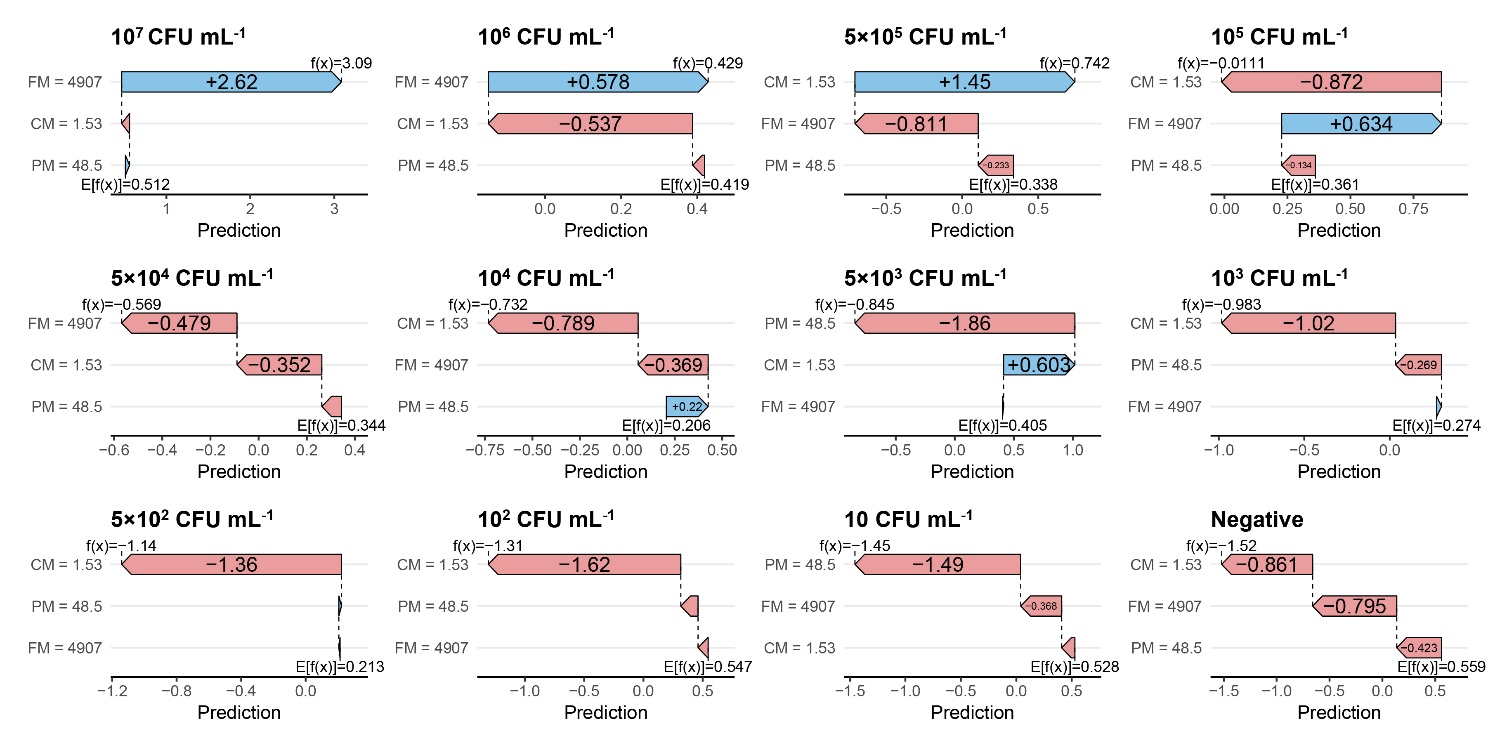
 **Figure S15.** Single sample interpretation of triple signals through waterfall plots for *S. typhimurium* detection.

# Table S1. Comparison of Michaelis-Menten constants (K_m_), maximum initial reaction rates (V_max_), and nanozyme activity unit (SA) of APzyme and some previous mimetics.

| **Catalysts** | **K_m_ (mM)** | | **V_max_** **(μM s^-1^)** | | **SA (U mg^-1^)** | **Reference** |
| --- | --- | --- | --- | --- | --- | --- |
|  | **TMB** | **H_2_O_2_** | **TMB** | **H_2_O_2_** |  |  |
| AFRNBs@PtNPs | 0.0984 | 2.576 | 0.444 | 0.159 | 17.75 | This work |
| PtNPs | 0.669 | 36.027 | 0.533 | 0.459 | 5.32 | This work |
| Ptclusters_2.1_ | 0.4964 | 0.9504 | 0.0223 | 0.0191 | 8.51 | ^6^ |
| Ptclusters_3.2_ | 0.4394 | 1.183 | 0.0266 | 0.0278 | 13.5 | ^6^ |
| Ptclusters_4.0_ | 0.6488 | 2.900 | 0.0394 | 0.100 | 16.3 | ^6^ |
| Pd@IrNSs | 0.01797 | 7.4831 | 0.168 | 1.208 | 7.44 | ^7^ |
| Ti_3_C_2_T*_x_*-Pt-PEG | / | 34.91 | / | 0.0722 | 1.78 | ^8^ |
| PtNi NWs | 0.275 | 14.73 | 35.53 | 35.56 | 10.43 | ^9^ |
| Pt NWs | 0.218 | 73.04 | 13.05 | 32.85 | 3.31 | ^9^ |
| BSA–Os | 0.155 | 7.61 | 1.45 | 1.65 | 6.12 | ^10^ |
| Pt_50_Sn_50_-PEG | / | 74.13 | / | 0.137 | 2.28 | ^11^ |
| Fe-Cu/ATP | 0.70 | 1.30 | 0.266 | 0.102 | 0.304 | ^12^ |
| PBCT^ER^ | 4.66 |  | 0.0154 |  | 0.363 | ^13^ |
| LiFeO_2_-VMT | / | 0.02 | / | 0.0364 | 7.9 × 10^–3^ | ^14^ |
| Fe-Doped MoS_2_ | / | 0.030 | / | 0.0201 | / | ^15^ |

# Table S2 Comparison of the developed nano-ELISA with previous works to detect *S. typhimurium*.

| Catalysts | Readout Mode | Substrates | LOD (CFU mL^-1^) | Linear Range (CFU mL^-1^) | Reference |
| --- | --- | --- | --- | --- | --- |
| Carbon Nanotubes-HRP | Colorimetric | TMB | 10^4^ | / | ^16^ |
| HRP | Colorimetric | TMB | 10^4^ | / | ^17^ |
| HRP | Chemiluminescent | Luminol | 3.63 × 10^3^ | 5.1×10^3^-1.2×10^6^ | ^18^ |
| Time-resolved fluorescent -PtNPs | Colorimetric | TMB | / | 5×10^3^-10^5^ | ^19^ |
|  | Fluorescent | / | 50 | 10^3^-10^5^ |  |
| MIL-88@Pd/Pt | Colorimetric | TMB | 32 | 40-4×10^5^ | ^20^ |
| MoS_2_@Au | Colorimetric | TMB | 10^2^ | / | ^21^ |
| HRP | Colorimetric | TMB | 1637 | 10^4^-10^7^ | This work |
| AFRNBs@PtNPs | Colorimetric | TPEN | 220 | 10^2^-10^6^ | This work |
|  | Fluorescent |  | 204 | 10^2^-10^6^ |  |
|  | Photothermal |  | 299 | 10^2^-10^6^ |  |

# References

1. Kresse, G.; Hafner, J., Ab initio molecular dynamics for liquid metals. *Physical review B* **1993,** *47* (1), 558.

2. Kresse, G.; Hafner, J., Ab initio molecular-dynamics simulation of the liquid-metal–amorphous-semiconductor transition in germanium. *Physical Review B* **1994,** *49* (20), 14251.

3. Perdew, J. P., Generalized gradient approximation made simple. *Phys. Rev. Lett.* **1997,** *77*, 3868.

4. Kresse, G.; Joubert, D., From ultrasoft pseudopotentials to the projector augmented-wave method. *Physical review b* **1999,** *59* (3), 1758.

5. Blöchl, P. E., Projector augmented-wave method. *Physical review B* **1994,** *50* (24), 17953.

6. Chen, S.; Yu, Z.; Wang, Y.; Tang, J.; Zeng, Y.; Liu, X.; Tang, D., Block-polymer-restricted sub-nanometer Pt nanoclusters nanozyme-enhanced immunoassay for monitoring of cardiac troponin I. *Analytical chemistry* **2023,** *95* (38), 14494-14501.

7. Ye, Z.; Fan, Y.; Zhu, T.; Cao, D.; Hu, X.; Xiang, S.; Li, J.; Guo, Z.; Chen, X.; Tan, K., Preparation of two-dimensional Pd@ Ir nanosheets and application in bacterial infection treatment by the generation of reactive oxygen species. *ACS Applied Materials & Interfaces* **2022,** *14* (20), 23194-23205.

8. Zhu, Y.; Wang, Z.; Zhao, R.; Zhou, Y.; Feng, L.; Gai, S.; Yang, P., Pt Decorated Ti3C2T x MXene with NIR-II Light Amplified Nanozyme Catalytic Activity for Efficient Phototheranostics. *ACS nano* **2022,** *16* (2), 3105-3118.

9. Guo, J.; Dong, C.; Zhang, X.; Liu, Y.; Leng, Y.; Wang, G.; Chen, Z., Colorimetric sensors constructed with one dimensional PtNi nanowire and Pt nanowire nanozymes for Hg2+ detection. *Analytica Chimica Acta* **2024,** *1321*, 343039.

10. He, S.-B.; Lin, M.-T.; Yang, L.; Noreldeen, H. A.; Peng, H.-P.; Deng, H.-H.; Chen, W., Protein-assisted osmium nanoclusters with intrinsic peroxidase-like activity and extrinsic antifouling behavior. *ACS Applied Materials & Interfaces* **2021,** *13* (37), 44541-44548.

11. Zhu, Y.; Zhao, R.; Feng, L.; Wang, C.; Dong, S.; Zyuzin, M. V.; Timin, A.; Hu, N.; Liu, B.; Yang, P., Dual nanozyme-driven PtSn bimetallic nanoclusters for metal-enhanced tumor photothermal and catalytic therapy. *ACS nano* **2023,** *17* (7), 6833-6848.

12. Feng, F.; Zhang, X.; Mu, B.; Wang, P.; Chen, Z.; Zhang, J.; Zhang, H.; Zhuang, J.; Zhao, L.; An, Q., Attapulgite doped with Fe and Cu nanooxides as peroxidase nanozymes for antibacterial coatings. *ACS Applied Nano Materials* **2022,** *5* (11), 16720-16730.

13. Xie, Y.; Wang, M.; Qian, Y.; Li, L.; Sun, Q.; Gao, M.; Li, C., Novel PdPtCu Nanozymes for Reprogramming Tumor Microenvironment to Boost Immunotherapy Through Endoplasmic Reticulum Stress and Blocking IDO‐Mediated Immune Escape. *Small* **2023,** *19* (44), 2303596.

14. Tian, L.; Qian, Y.; Wang, H.; Zhao, G.; Tang, A.; Yang, H., Mineral Phase Reconfiguration Enables the High Enzyme-like Activity of Vermiculite for Antibacterial Application. *Nano Letters* **2023,** *24* (1), 386-393.

15. Ali, S. R.; De, M., Fe-doped MoS2 nanozyme for antibacterial activity and detoxification of mustard gas simulant. *ACS Applied Materials & Interfaces* **2022,** *14* (38), 42940-42949.

16. Chunglok, W.; Wuragil, D. K.; Oaew, S.; Somasundrum, M.; Surareungchai, W., Immunoassay based on carbon nanotubes-enhanced ELISA for Salmonella enterica serovar Typhimurium. *Biosensors and Bioelectronics* **2011,** *26* (8), 3584-3589.

17. Wang, W.; Liu, L.; Song, S.; Tang, L.; Kuang, H.; Xu, C., A highly sensitive ELISA and immunochromatographic strip for the detection of Salmonella typhimurium in milk samples. *Sensors* **2015,** *15* (3), 5281-5292.

18. Zhang, C.; Liu, Z.; Bai, M.; Wang, Y.; Liao, X.; Zhang, Y.; Wang, P.; Wei, J.; Zhang, H.; Wang, J., An ultrasensitive sandwich chemiluminescent enzyme immunoassay based on phage-mediated double-nanobody for detection of Salmonella Typhimurium in food. *Sensors and Actuators B: Chemical* **2022,** *352*, 131058.

19. Hu, J.; Chen, C.-H.; Wang, L.; Zhang, M.-R.; Li, Z.; Tang, M.; Liu, C., Multi-functional nanozyme–based colorimetric, fluorescence dual-mode assay for Salmonella typhimurium detection in milk. *Microchimica Acta* **2024,** *191* (8), 464.

20. Yuan, J.; Duan, H.; Wang, L.; Wang, S.; Li, Y.; Lin, J., A three-in-one hybrid nanozyme for sensitive colorimetric biosensing of pathogens. *Food Chemistry* **2023,** *408*, 135212.

21. Lu, L.; Ge, Y.; Wang, X.; Lu, Z.; Wang, T.; Zhang, H.; Du, S., Rapid and sensitive multimode detection of Salmonella typhimurium based on the photothermal effect and peroxidase-like activity of MoS2@ Au nanocomposite. *Sensors and Actuators B: Chemical* **2021,** *326*, 128807.
